# Nanoparticles in Dentistry: A Comprehensive Review

**DOI:** 10.3390/ph14080752

**Published:** 2021-07-30

**Authors:** Gustavo Moraes, Carolina Zambom, Walter L. Siqueira

**Affiliations:** 1 College of Dentistry, University of Saskatchewan, Saskatoon, SK S7N 5E4, Canada; moraes.gustavo29@yahoo.com.br; 2 Department of Dentistry, State University of Ponta Grossa, Ponta Grossa 84030-900, Brazil; 3 Department of Biochemistry and Organic Chemistry, Institute of Chemistry, UNESP—Sao Paulo State University, Araraquara 14800-060, Brazil; carolinarz@gmail.com

**Keywords:** nanoparticles, drug delivery, dental caries, periodontal diseases, dental prosthesis, head and neck neoplasms, hyposalivation

## Abstract

In recent years, nanoparticles (NPs) have been receiving more attention in dentistry. Their advantageous physicochemical and biological properties can improve the diagnosis, prevention, and treatment of numerous oral diseases, including dental caries, periodontal diseases, pulp and periapical lesions, oral candidiasis, denture stomatitis, hyposalivation, and head, neck, and oral cancer. NPs can also enhance the mechanical and microbiological properties of dental prostheses and implants and can be used to improve drug delivery through the oral mucosa. This paper reviewed studies from 2015 to 2020 and summarized the potential applications of different types of NPs in the many fields of dentistry.

## 1. Introduction

Nanotechnology has become one of the most active research areas in the past decades, especially in health sciences [1]. Nanoparticles (NPs) are discrete clusters of atoms with a wide range of medical applications, including cancer therapy, drug delivery, tissue engineering, regenerative medicine, biomolecules detection, and also as antimicrobial agents [2]. NPs are generally classified into organic (dendrimers, micelles, liposomes, or polymers), inorganic (metal or metal oxide based), or carbon = based (fullerenes, graphene, or carbon nano tubes) [3].

One of the main challenges for dental researchers is to develop materials that can withstand the harsh conditions of the oral environment while remaining biologically sustainable and biocompatible [4,5]. NPs are gaining momentum in dentistry due to their physicochemical and biological properties, including biocompatibility, size, charge, large surface area, strength, solubility, chemical and surface reactivity, color, high stability, and thermal conductivity [6,7,8]. Such properties have allowed the development of new, innovative materials and the expansion and improvement of their functions [9]. Despite having countless advantages, some NPs also have limitations, including toxicity, limited delivery, and being difficult to handle [8].

Considering the aforementioned and the recent advances in this field, we reviewed studies published from 2015 to 2020 and summarized the potential applications of different types of NPs in the diagnosis, prevention, and/or treatment of the most common oral diseases. We also discussed how these NPs can enhance the physicochemical and biological characteristics of dental implants and removable prostheses, and how they can improve the delivery of drugs via the buccal route.

## 2. Dental Caries

Dental caries is the most prevalent oral diseases among the population worldwide. For this reason, it is considered a public health problem, making the development of alternative treatments imperative [10]. Dental caries is a multifactorial disease and is usually caused by microbial colonization of the dental surface and the formation of biofilms. The microorganisms present in the oral cavity promote sugar metabolism generating acidic metabolites that drastically reduce the osral pH [10,11]. The acidification of the pH starts the process of demineralization of the teeth, that leads to the loss of calcium, fluoride, and phosphate ions, resulting in the formation of cavities [12].

This process can be reversed with fluoride application or by using toothpaste and mouthwashes containing fluoride. The use of NPs is an important tool and can help in this type of treatment [12,13]. NPs can be defined as ultra-dispersed supramolecular structures, with sizes from 10 µm to 1000 µm [14]. Drugs can be encapsulated, dissolved, trapped, or attached to the structure of NPs and this can be very useful in dentistry. For example, NPs with adequate amounts of fluoride can be applied in the oral cavity to increase fluoride levels. This application allows the remineralization of the teeth and avoids the process of caries formation [12]. Calcium fluoride nanoparticles (CaF_2_NPs) have been widely used to increase the level of fluoride present in the oral cavity. According to Kulshrestha et al., CaF_2_NPs are capable of inhibiting the production of exopolysaccharide by *Streptococcus mutans* [15]. These NPs have a dual effect: to promote the remineralization of tooth enamel and to prevent the development of biofilm. However, the residence time in the oral cavity of CaF_2_NPs is short and, in order to improve this characteristic, the CaF_2_NPs can be loaded in chitosan bioadhesive films [12,16,17]. Chitosan is a polymer widely used for oral applications because of its mucoadhesion, biocompatibility, low toxicity, and controlled release of drugs. A study conducted by Ghafar et al. incorporated CaF_2_NPs in a bioadhesive chitosan film and the CaF_2_NPs remained intact for up to 3 h. This approach provided an increase in the residence time of CaF_2_NPs in the oral environment, showing an advantage when compared to the use of toothpaste and mouthwashes, which remain for a few minutes in the oral cavity [12]. 

In addition to chitosan, pectin and alginate are examples of other polymers that also have mucoadhesion, biocompatibility, biodegradability, and low toxicity. For this reason, Nguyen et al. evaluated the capacity of these three polymers to produce nanoparticles loaded with sodium fluoride. The best results were obtained with chitosan NPs, while alginate was not able to form nanoparticles. Chitosan NPs showed slow and continuous fluoride release, with increased release in acidic conditions [13]. Despite requiring in vivo studies, chitosan was the best option for obtaining NPs and showed slow fluoride release. 

Another alternative for dental caries treatment is the use of restorative materials with bioactive functions [18]. The addition of chitosan NPs in a glass ionomer cement (GIC) is one of these strategies [18,19,20]. Chitosan NPs have bactericidal effect and provide fluoride release from the material, promoting a dual effect in the prevention of dental caries. Kumar et al. demonstrated that the addition of 10 wt.% of chitosan NPs in GIC increased the material resistance and fluoride release [18]. However, Ibrahim et al. showed that the addition of more than 25% (*v*/*v*%) of chitosan NPs in the GIC improved its bactericidal effect against *S. mutans*, but decreased its physical properties, leading to ruptures and poor adhesion to dentin [19]. For this reason, these same authors added titanium oxide NPs (TiO_2_NPs) to the GIC. The hybrid material showed antimicrobial activity with inhibition of biofilm growth. In addition, the TiO_2_NPs improved some physical characteristics, such as flexibility and compressibility [20].

In another study, Aliasghari et al. showed that chitosan NPs have anti-growth and anti-adherence effects against cariogenic bacteria in vitro [21]. Chitosan is a polymer with antimicrobial properties by itself. This potential can be increased by loading antibiotics in chitosan NPs [17,22]. Chitosan NPs are widely used in dentistry as hydrogels and nanofibers for application in the oral cavity [17,21,22]. The use of chitosan NPs is also reported by Ikono et al., who found antifungal action against *Candida albicans* [23]. Covarrubias et al. coated copper NPs with chitosan and proved that the bactericidal effect of the NPs against *S. mutans* increased and was equivalent to the traditional antimicrobials such as chlorhexidine and cetylpyridinium chloride [16]. Ren et al. produced a chitosan hydrogel incorporated with a peptide that promotes tooth remineralization. This peptide combined with chitosan hydrogel showed a dual effect with antibacterial effect and tooth remineralization properties [24]. 

Other polymeric NPs composed of poly(ethyleneglycol) (PEG) and polylactic-co-glycolic acid (PLGA) are used for the prevention and treatment of dental caries. These polymers are mucoadhesive, biocompatible, biodegradable, and suitable for promoting prolonged release [25,26,27]. Sebelemetja et al. produced polymeric NPs using PEG-PLGA that showed bactericidal and anti-biofilm effects against *S. mutans* [26]. Zhao et al. produced polymeric NPs loaded with chlorhexidine which were able to rapidly release the drug in acidic pHs [28]. The same strategy was also proposed by Liu et al. using the polymers PEG-b-PCL and PCL-b-PAE [25]. These polymeric NPs along with chitosan NPs and fluoride NPs are highly promising for dental caries therapy and can help to design other drug-delivery systems.

## 3. Periodontal Diseases

The cause of periodontal disease is the imbalance between the colonization of bacterial pathogens and the host immune response toward infection [29]. The first step in the development of periodontal disease is the formation of dental plaque and gingivitis, which consist in the inflammation of gingiva. The chronic stage occurs later, when the immune cells are recruited and activated. These cells release pro-inflammatory cytokines and reactive oxygen species (ROS) that cause alveolar bone and periodontal ligament destruction [29,30].

The use of NPs for the eradication of pathogenic bacteria is an effective approach to treat periodontal disease since bacterial colonization is one of the first steps to trigger this condition. Emmanuel et al. reported that silver nanoparticles (AgNPs) associated with azithromycin and clarithromycin have a synergistic antimicrobial efficacy against periodontal disease causing microorganisms [31]. The platinum nanoparticles (PtNPs) developed by Itohiya et al. have been shown to mediate antibacterial effects caused by *S. mutans*, *Enterococcus faecalis*, and *Porphyromonas gingivalis* [32]. These microorganisms are associated with dental caries, endodontic lesions, and periodontal diseases. Similarly, Vega-Jiménez et al. developed bismuth subsalicylate NPs with antibacterial effect against periodontal pathogens *Aggregatibacter actinomycetemcomitans*, *Capnocytophaga gingivalis*, and *P. gingivalis*, with low toxicity against human gingival fibroblast (HGF-1) cells [33]. In another study, Holden et al. produced glutathione-capped bimetallic NPs with great antibacterial potential against anaerobic oral pathogen *P. gingivalis* [34]. 

These nanoparticles can be used alone or in association with biomaterials to promote prolonged residence time in the oral cavity. Lee et al. loaded AgNPs into electrospun nanofibers to enhance its bio-functionality. This biomaterial has also been employed for oral drug delivery and for the development of an antimicrobial oral wound dressing to inhibit periodontitis and gingivitis [35]. Another example of biomaterial for dental application is the gelatin and chitosan composite guided tissue regeneration membrane containing hydroxyapatite nanoparticles and antimicrobial peptide (Pac-525)-loaded PLGA microspheres developed by He et al. This drug delivery system showed sustained release and antibacterial activity for a long period of time against *S. aureus* and *Escherichia coli* [36]. To promote a rapid release of an antibacterial peptide (BAR), Mahmoud et al. developed polymeric electrospun fibers to encapsulate it. The development of this platform could rapidly release the peptide and disrupt the dual-species biofilm formed by *P. gingivalis* and *Streptococcus gordonii* [37].

Another method to prevent periodontitis is to treat the tissue inflammation caused by the chronic form of the disease. Nitric oxide-releasing silica NPs [38], polydopamine NPs [29], and silica NPs [30] are examples of NPs that used anti-inflammatory compounds for treating periodontal disease. After treatment with some of these NPs, it was possible to observe a decrease in local periodontal inflammation and a higher antioxidant activity [29,30]. The metformin hydrochloride-loaded PLGA NPs produced by Pereira et al. also controlled inflammation and bone loss in an experimental periodontal disease model by managing to control blood glucose levels below what is considered diabetes [39]. In fact, prevention of bone loss and promotion of tissue repair are important factors for the treatment of chronic cases of periodontal disease. Mou et al. produced albumin microspheres containing minocycline and zinc oxide nanoparticles (ZnONPs) and observed gingival tissue self-repairing, antimicrobial activity, low toxicity, and high security at certain concentrations of ZnONPs [40]. Osorio et al. observed periodontal regeneration by using calcium- and zinc-loaded NPs. These NPs can promote precipitation of calcium phosphate deposits and were found to be non-toxic against oral mucosa fibroblasts [41]. Despite the aforementioned promising results, further studies are needed to investigate these novel formulations with NPs, especially in vivo studies and controlled clinical trials.

Kalia et al. used polymeric NPs to deliver the BAR peptide to inhibit *P. gingivalis* and *S. gordonii* biofilm formation. These authors noticed a higher local dose of peptide compared to treatment with formulations of free peptide. The BAR-modified NPs also disrupted the preformed biofilms more effectively [42]. Zambrano et al. found that curcumin-loaded polymeric NPs may reduce inflammation and the connective tissue destruction associated with periodontal disease [43]. In addition, Wijetunge et al. found that wheat germ agglutinin liposomes loaded with ciprofloxacin and betamethasone promote potent antibacterial and synergistic anti-inflammatory effects for up to 24 h [44]. Liposomes were used by Moraes et al. to promote pain control during scaling and root planning treatment. The liposomes loaded with lidocaine/prilocaine could be a good option to increase patient compliance during this type of clinical treatment, especially anxious patients or those who have a fear of needles [45].

## 4. Pulp and Periapical Lesions

Exposure of the dental pulp is a challenging problem to treat successfully, regardless of the cause [46]. The primary goal of endodontic therapy is to eliminate microbial infection and promote periapical tissue healing [47]; however, complete eradication of intracanal microbial load is considered practically impossible and has yet to be achieved [48,49]. Endodontics can benefit from the emergence of nanotechnology due to its broad-spectrum antibacterial and anti-biofilm activity and biocompatibility [50].

Chitosan has many applications in endodontics. It can be successfully used as a chelating substance [51,52,53,54,55], as a scaffold for the delivery of growth factors [56], or medications [57]. Moreover, it can be used as an irrigant solution [47,58,59,60] with less toxicity than sodium hypochlorite or chlorhexidine [58,59], as an intracanal medication by itself [61], or as an adjunct to calcium hydroxide [62,63]. Chitosan can increase the antibacterial efficacy, bond strength, and penetration of root canal sealers, directly or indirectly [48,64,65,66]. In addition, some studies suggest that chitosan could be used as an auxiliary for procedures such as apexification [67], endodontic retreatment [68], pulp-capping [46,69,70,71], and pulpotomy [72], although it was not able to induce apexogenesis in immature necrotic permanent teeth [73] or to form new mineralized tissues along the root canal walls of immature dog teeth with apical periodontitis [74].

AgNPs are also widely used in this field. It has been shown that these NPs, combined or not with other substances, are effective against *E. faecalis* [75,76,77,78] and other endodontic-periodontal pathogens [79], are biocompatible [80], have low cytotoxicity and genotoxicity [81], and can be used as endodontic irrigants [75,82], chelating agents [83], root canal sealers [84], and/or repair cements [85]. However, some authors have reported that AgNPs can cause tooth discoloration when used as an intracanal medicament [86], while others have claimed otherwise [50]. Perhaps, the combination of AgNPs with calcium hydroxide might be the reason for these contrasting results, as it did not promote significant changes in tooth color [50].

Several different NPs have been investigated for endodontic therapy. The obturation properties of gutta percha can be enhanced with diamond NPs [87]. A randomized clinical trial with six months of follow-up reported that there was a decrease in the size of periapical lesions after obturation with modified gutta percha, with no adverse events [87]. Gold (Au) and iron oxide NPs can inhibit pathogenic biofilm formation [88,89] and invasion to dental pulp stem cells [88]. Some studies proposed propolis-loaded NPs of PLGA and NPs of amorphous calcium phosphate as endodontic sealers, given their antimicrobial activity [90,91], good bond strength to root canal [90], and/or cytocompatibility [91]. PLGA-moxifloxacin NPs and chlorhexidine hydrochloride nanoemulsions could be employed as irrigant solutions, considering their antibacterial activity against *E. faecalis* [6,92], cleansing ability [92], and/or sustained release [6]. Nanotechnology can also be used to promote pulp tissue repair and regeneration [93], a topic of great interest in dentistry.

NPs can have their properties improved by being conjugated with other substances and materials. The combination of photodynamic therapy and NPs can reduce the presence of *E. faecalis* in root canals [94,95,96] and remove the smear layer from the apical third of root canals [97]. The association of chitosan and EDTA can simultaneously disinfect root canals and remove the smear layer [98].

Despite the aforementioned advantages, NPs can also have some limitations. For example, one randomized, double-blind, clinical trial did not find significant differences between a liposomal bupivacaine formulation and bupivacaine alone in the management of symptomatic patients with pulpal necrosis experiencing moderate to severe preoperative pain [99]. Another clinical trial from the same group did not find significant differences between the same liposomal bupivacaine formulation and 2% lidocaine with 1:100,000 epinephrine for pain reduction in untreated symptomatic irreversible pulpitis [100], indicating that there are some situations where NPs are not as effective as the free, conventional drug.

## 5. Peri-Implantitis and Implant Failures

Implant-prosthetic rehabilitation has become the preferred therapy for tooth loss and the gold standard in modern dentistry because of its ability to integrate with the surrounding alveolar bone [101,102,103]. Peri-implantitis is one of the most common causes of implant loss and is caused by a microbial biofilm surrounding the surface of the implant that leads to inflammation [104].

Many NPs have been studied aiming to reduce surface contamination and the occurrence of peri-implantitis. Studies have shown the antimicrobial activity of NPs against pathogens related to peri-implantitis, including *S. gordonii* [104], *S. mutans* [105,106], *P. gingivalis* [107,108], *Staphylococcus aureus* [109], *C. albicans* [110], *E. coli* [111,112], *Streptococcus sanguinis*, and *A. actinomycetemcomitans* [113]. NPs can prevent or treat the infection through alternative means, such as by using a chitosan brush to perform the debridement [114,115]; by applying a chitosan-based thermosensitive hydrogel, which reportedly has antibacterial properties and could act as a lubricant and sealant simultaneously [116]; by delivering drugs or proteins to the target tissue via a liposome-modified titanium surface [117]; by brushing the implant surfaces with an implant-paste based on two-dimensional nanocrystalline magnesium phosphate gel and hydrated silica NPs [101]; or by coating the implant surface with a chitosan matrix containing gelatin nanospheres loaded with antibiotics [118]. 

Inorganic NPs have many different roles in periodontology, especially in the prevention and treatment of peri-implantitis. Copper and AgNPs present antibacterial properties [119,120,121,122], AuNPs can be used as a bone inductive adjuvant [103], bismuth NPs can improve the treatment of peri-implantitis and peri-implant mucositis [122], titanium NPs prevent fungal and bacterial adhesion to the implant surface [123,124], and hydroxyapatite NPs can be combined with AgNPs, aiming to enhance their biocompatibility [125].

The clinical success of titanium implants depends on their surface characteristics, which can influence cell adhesion, proliferation, differentiation, and their integration with surrounding tissues [126,127]. Using hydroxyapatite nanocrystals to treat titanium surfaces can increase cell proliferation, differentiation, and spread and contribute to the synthesis of bone matrix, and consequently, osseointegration [126]. Hydroxyapatite NPs seem to be an excellent scaffold for bone implant integration, considering the high cell adhesion and osteoblast viability presented in the study conducted by De Lima Cavalcanti et al. [127]. Hydroxyapatite NPs can also be combined with chitosan and graphene oxide to fabricate a composite coating, which greatly heightened the cell–material interactions in vitro and enhanced osseointegration in vivo [128]. AuNPs were used to coat titanium surfaces, which showed significantly enhanced osteogenic differentiation and influence on the osseous interface formation [129].

Chitosan has many applications in this field, as it can be used to coat surfaces in order to increase their biocompatibility and bioactivity [130]. Chitosan can also be conjugated with other substances, including AgNPs [131,132], hyaluronic acid [131,133,134], poly(dopamine) and hydroxyapatite [135], collagen [136], silica [137], and poly(acrylic acid) [138]. These substances, alongside chitosan, can prevent the surface adhesion of bacteria [131,132,133,134,138], increase the proliferation of cells [137,138], and improve osseointegration [134], soft tissue integration [135], and peri-implant tissue attachment [136].

NPs can also be used for sinus floor elevation and augmentation procedures. Nanocrystalline and nanoporous hydroxyapatite were tested during a clinical study and proved to support bone formation, with no clinical and histological signs of inflammation in the augmented sites [139]. The use of hydroxyapatite NPs for augmentation with simultaneous implant placement was compared to a graftless tenting technique in another clinical trial, and both showed successful results regarding implant stability [140].

Patients with special needs can benefit from the advances of nanotechnology as well. Chitosan-AuNPs facilitated PPARγ gene delivery on dental implants and contributed to osseointegration, new bone formation, and mineralization in diabetic-induced rats, suggesting it can be used as a therapeutic approach in diabetic patients. This gene consists of transcription factors and is closely linked to the metabolism of glucose homeostasis [141]. Chitosan-AuNPs were also able to deliver c-myb, a transcription factor involved in the control of cell proliferation, differentiation, survival, and death, to the target area and consequently enhance bone formation in the osteoporosis [142]. Another option for osteoporotic patients might be implants coated with gelatin/chitosan and insulin-like growth factor 1, a strategy that promoted osseointegration in osteoporotic conditions both in vivo and in vitro [143]. A liposomal bupivacaine formulation might help those who are more sensitive to pain to experience a significant reduction of postsurgical pain and opioid consumption [144].

## 6. Dental Prosthesis Failures

Although dental implants are becoming more accessible and affordable, in many cases, for medical and financial reasons, a conventional denture is still the preferred treatment for edentulous patients [145,146].

Dentures are usually made with conventional heat-polymerized polymethylmethacrylate (PMMA) [147] due to its biocompatibility, esthetics, stability in the oral environment, ease of repair, tasteless and odorless properties, high polishability, low cost, acceptability by the patients, light weight, and low water sorption and solubility levels [145,148,149,150]. However, PMMA has poor mechanical properties, such as low flexural and impact strength, low fracture resistance, insufficient surface hardness, fatigue failure, and surface roughness, which allows microbial adhesion [147,149,151,152,153,154]. 

Given the aforementioned limitations of conventional dental prostheses and the fact that PMMA is a clear polymer capable of being modified, new technologies have been developed in order to improve its physicochemical properties [147]. The ideal material must balance its achieved properties while still managing to be biocompatible, readily available, cost effective, antimicrobial, easy to manipulate, capable of maintaining sufficient bond strength with artificial teeth and lining materials, functionally efficient, and esthetically pleasing [150,155,156]. The incorporation of organic and inorganic NPs into acrylic resins has been proposed with this aim.

Zirconium oxide (ZrO_2_) NPs were one of the most common types of NPs tested in this field in the past years. The addition of ZrO_2_NPs can significantly increase the dimensional accuracy, decrease the impact strength [155], and increase the tensile strength of the denture base acrylic [150], while the translucency of the PMMA can be reduced as the concentration of nano-ZrO_2_ increases [150]. Incorporation of ZrO_2_NPs can also improve the flexural and transverse strength and reduce *Candida* adhesion to repaired denture bases [157,158,159]. By mixing these NPs with glass fibers, the flexural and impact strengths of PMMA can significantly improve [149]. 

The effect of incorporating silica NPs into PMMA has also been described in the literature. The silica incorporation into acrylic resin decreased its flexural strength when compared to PMMA alone [148]. Furthermore, Karci et al. found lower flexural strength values in the groups with silica NPs when compared with those of titanium and aluminum NPs [152]. When silica aluminum borate whiskers were combined with Novaron AG300, tetra-needle-shaped zinc oxide whisker, and silanized ZrO_2_NPs, the composites presented substantially higher antibacterial activity, flexural strength, and surface hardness, without compromising their cytotoxicity [153]. By silanizing SiO_2_NPs with γ-methacryloxypropyltrimethoxysilane, the PMMA specimens presented adequate flexural strength, flexural modulus and fracture toughness, with a clinically acceptable color [160].

Zinc oxide (ZnO) NPs have also been receiving attention recently. In some concentrations, these NPs can present antifungal properties [9,161], increase the hardness, thermal stability, glass transition temperature, and the hydrophilicity of PMMA composites, and do not compromise the properties of acrylic resin in a way that could disqualify its clinical use [162,163]. However, some studies report the opposite, stating that ZnONPs are not capable of inhibiting biofilm formation nor increasing the glass transition temperature [164]. When silanized with methacryloxypropyltrimethoxysilane, ZnONPs can present greater antifungal effect, fewer color differences, and opacity compared to nonsilanized NPs [165].

Moreover, other NPs have been investigated. The addition of nanodiamonds to acrylic denture base improved its flexural strength and surface roughness at low concentrations, but its impact strength was compromised [147]. Prepolymer incorporation resulted in increased flexural strength of acrylic resins when compared to silica addition [148]. The incorporation of AgNPs affected the transverse strength of the denture base acrylic resins in a concentration-dependent manner and decreased the glass transition temperature in the study conducted by Koroglu et al. [154]. The concentration of AuNPs added to PMMA can have significantly different effects on PMMA flexural strength [151]. The addition of titanium dioxide NPs can considerably modify the acrylic resin color and decrease its flexural strength, but it can also significantly increase the impact strength, tensile strength, and microhardness, depending on the concentration of NPs [166,167].

Regarding the prevention of denture-related infections, incorporation of titanium dioxide or AgNPs in PMMA was proved to have antimicrobial activity, especially in higher concentrations and against *Candida* species [145,168,169]. High concentrations, however, could result in lower mechanical parameters [145]. The addition of nanostructured Ag vanadate can provide acrylic resins with antibacterial activity but reduces their impact strength [170]. By adding nano-chitosan particles to acrylic resins, biofilm formation of *Candida* species can be significantly reduced [171]. The incorporation of platinum and diamond NPs into a silica coating agent can improve its hydrophilicity and longevity, helping prevent microorganisms from adhering to the denture surface [156].

## 7. Oral Candidiasis and Denture Stomatitis

Oral candidiasis is an opportunistic fungal infection that affects approximately 2 million people around the world annually, especially those who are immunocompromised [171,172,173], and it is characterized by patches of creamy white exudate with a reddish base covering the mucous membrane of the tongue, cheeks, palate, and oropharynx [173]. Denture stomatitis, on the other hand, manifests as palate and alveolar ridge erythema [174] and it is the most prevalent form of oral candidiasis [175]. 

Treating oral candidiasis and denture stomatitis can be challenging, as it involves the improvement of oral and/or denture hygiene, relining or replacing the prostheses, avoiding overnight use of dentures, and the topical and/or systemic administration of antifungal drugs [174]. However, the effectiveness of treatment can be compromised by the cost of medication [176], its unpleasant taste, toxicity, and possible side effects, including nausea, mouth irritation, vomiting, and diarrhea [173,174], poor oral absorption [177], drug resistance [176], and the cleansing effect of saliva [173]. Nanotechnology has been proposed to overcome such obstacles, given that unconventional antimicrobial agents hold great potential in the treatment of infectious diseases [178].

The powerful fungicide effect of chitosan and its capacity to inhibit *C. albicans* adhesion and biofilm formation have been described by many authors [179,180,181,182]. Fabio et al. evaluated the possible enhancive effect of chitosan on the photosensitizer methylene blue. Although chitosan has a strong antifungal action, it does not improve the methylene blue activity during photodynamic therapy [183]. The effect of other NPs combined with antimicrobial photodynamic therapy have also been assessed. Cationic curcumin-polymeric NPs can reduce *C. albicans*, even in the absence of light [184], and chloro-aluminum phthalocyanine encapsulated in cationic nanoemulsions seems to be an effective photosensitizer agent to use [185].

The efficacy of chitosan along with its inherent biocompatibility also makes it a promising candidate for use as a mouthwash [174]. Some authors have developed mouthwashes with chitosan alone [174] or in combination with curcumin [186,187]. Chitosan mouthwash significantly decreased the erythematous area, burning sensation, time required for clinical improvement, and number of blastospores and mycelia in a clinical trial [174], while the combination with curcumin presented a favorable clinical response with no local or systemic adverse events neither in animals [186], nor in humans [187]. 

Mucoadhesives, gels, toothpastes, buccal tablets, and buccal films have been proposed as treatments for oral candidiasis. Such formulations were developed with NPs for improved local delivery of drugs like miconazole [176,188,189,190], nystatin [173,177], amphotericin B [191], and fluconazole [192], or plant-derived products, such as stigmasterol [193] or *Glycyrrhiza glabra* L. extract [194]. Some of these formulations presented enhanced antifungal activity, reduced cytotoxicity, faster drug release rate, improved dissolution rate, and better prolonged release than marketed products or the non-loaded drug [176,188,189,190,191], while others, despite not being compared to commercialized products, have shown promising results [173,177,192,193,194].

One alternative therapy for denture stomatitis is the incorporation of NPs, such as chitosan and ZnO-Ag, into tissue conditioners or soft liners. These modified materials present a significant antifungal and antibacterial activity and are not cytotoxic [195,196,197,198]. The incorporation of such NPs into tissue conditioners or soft liners did not compromise some mechanical properties, such as shore A hardness, surface roughness, and tensile bond strength, in a clinically relevant manner [195,199]. Chitosan has also shown properties suitable for development into an antifungal denture adhesive that could inhibit *C. albicans* adherence to denture base acrylic resin [200].

NPs have been employed to protect antimicrobial peptides from degradation in the oral cavity as well. Histatin 5 was encapsulated by liposomes [201] or combined with amphotericin B and coated with chitosan [202], with very positive results. Antimicrobial molecules cathelicidin LL-37 and ceragenin CSA-13 were developed with magnetic NPs and presented high antifungal activity and biocompatibility, resistance to inhibitory factors present in body fluids, and effective inhibition of fungal biofilm formation [178].

## 8. Head, Neck, and Oral Cancer

Around half a million people are diagnosed with oral cancer worldwide, and approximately 150,000 patients pass away yearly [203]. Early and accurate diagnosis allows a proper treatment, resulting in less morbidity and better prognosis [204]. However, treatment is usually challenging due to late diagnosis, high risk of invasion, rapid metastasis, frequent relapses, and painful side effects [205,206]. Considering the adverse effects of traditional anticancer treatment and their impact in the quality of life of the patients, nanotechnology has a great potential to improve early diagnosis and therapy [205].

A wide variety of NPs were proven to be effective against a number of oral cancer cell lines, including human Caucasian dysplastic oral keratinocytes (DOK) [205,207], mouth epidermoid carcinoma (KB) [203,208,209,210,211,212], murine AT-84 oral squamous carcinoma cells [213], oral squamous cancer cell lines of Asian origin (ORL-48 and ORL-115) [214], human oral squamous cell carcinoma lines (PE/CA-PJ15 [215], OEC-M1 [216], HSC2 [217], YD-9 [218], SAS [219,220], HSC4 [1,220], KOSC [220], HSC3 [204,221,222], HSC-3-M3 [7], Ca9-22 [223], CAL 27 [222,223,224,225,226,227], SCC131 [228], SCC4 [222,228], VB6 [229], and H357 [229,230,231]), human tongue carcinoma cell lines (SCC-9 [232,233,234], SCC-25 [224,230,233,235], SCC-15 [236], and SCC-090 [237]), and multidrug-resistant oral carcinoma cells (KB-Ch^R^-8-5) [238]. Despite the promising results, most of these studies were conducted in vitro, thus, further in vivo studies are necessary to confirm the efficacy of these nanomaterials.

Doxorubicin (DOX) and paclitaxel are two of the most commonly used chemotherapeutic drugs [239]. Taking their adverse effects into consideration, studies have proposed alternative forms of drug delivery, such as liposome formulations [239], solid lipid NPs [240], mucoadhesive alginate pastes with embedded liposomes [241], methotrexate-loaded stimuli responsive silica-based NPs [242], polymers [243], PEGylated NPs [244,245], mesoporous silica NPs-polymerpolyethylenimine [246], multifunctional targeted polymeric NPs [247], and a multifunctional Au nanoplatform [248]. In comparison to the free drugs, these new systems showed a higher percentage of apoptotic cells [239,247], fewer hematological changes [242], significant therapeutic efficacy [243,248], significant inhibitory effects on tumor growth [245], lower cytotoxicity and systemic toxicity, and higher tumor accumulation of DOX [244].

AuNPs and nanorods have many applications in diagnosis and tumor margin determination. These nanomaterials have been associated with different diagnostic tools and systems, including air scanning electron microscopy [249], enzyme-linked immunosorbent assay (ELISA) [250], optical coherence tomography [251], surface enhanced Raman spectroscopy (SERS) [252,253,254], an electrochemical immunosensor [255], indirect computed tomography and magnetic resonance lymphography [256], and a biosensor consisting of an upconversion nanoparticle, MMP2-recognized polypeptides, and quenchers [257]. Such methods were proven to be reproducible [250], sensitive [250,257], specific [250,255,257], stable [255], accurate [253], reliable [252], and/or capable of detecting early-stage cancer [251]. 

Other NPs can be used with similar aims: carbon NPs were able to track occult lingual lymph nodes in early-stage tongue squamous cell carcinoma with accuracy [258]; AgNPs were used as a substrate for SERS, which showed specificity and sensitivity above 90% [259]; nanostructured zirconia on reduced graphene oxide exhibited a wide linear detection range, excellent sensitivity, and a remarkable low detection limit when used as an immunosensor [260]; a SERS substrate was made up of leaf-like titanium oxide nanostructures decorated with AgNPs and presented rapid and accurate detection of solid tumors [261]; and nanostructured yttrium oxide was used for the fabrication of a biosensing platform, which showed a high linear range and remarkable sensitivity [262].

Head and neck squamous cell carcinoma is the sixth most common cancer worldwide [263]. Considering the side effects of chemo and radiotherapy, it is necessary to develop more effective and safe treatments [264]. Several NPs can be employed in the treatment of head and neck cancer, as they reportedly can enhance the efficacy of treatment [263], reduce or inhibit tumor growth [264,265,266,267], increase overall survival in animals [266], enhance tumor radiosensitization [265], suppress metastasis [268], induce cell apoptosis [206,264,269] and cell cycle arrest [269], and/or increase antiproliferative bioactivity [269]. AuNPs can be combined with X-ray irradiation to induce apoptosis [270], and PDT can be combined with lipid–calcium–phosphate NPs to deliver a vascular endothelial growth factor in order to decrease tumor volume [271].

Oral mucositis is one of the most common side effects induced by high-dose chemotherapy and/or radiation [272]. It not only compromises the quality of life of patients, but also affects compliance to treatment [272]. Studies suggest that different types of nanoformulations can be used to assess the risk of and treat chemotherapy- and radiotherapy-induced oral mucositis, including chitosan [272,273,274], PLGA [272], and hydroxypropyl methylcellulose [274]. These NPs can potentially be used in the development of muco-adhesive wound dressing materials [273] or sustained drug release systems [272,274].

## 9. Hyposalivation

Hyposalivation is the condition of having reduced or insufficient saliva production [275,276]. The use of some medication or radiation therapy for head and neck cancer treatment and autoimmune disorders (e.g., Sjogren’s syndrome) can lead to hyposalivation [276,277]. There is a clinical concern that hyposalivation decreases oral health and overall health in many patients [277]. Oral lesions, dental caries, dental demineralization, periodontal disease, and fungal infections are some of the consequences caused by the decrease of salivary flow [278]. It also contributes to difficulties in speaking, chewing, and swallowing [279]. 

Current treatments for hyposalivation are limited to medications such as the muscarinic receptor agonists, pilocarpine and cevimeline [276,277]. Therefore, the development of new therapeutics is essential for the prevention and treatment of this condition. It is also important to create alternatives to provide relief from dry mouth condition, one of the consequences of hyposalivation, in order to improve the quality of life of patients with this problem. The use of nanoparticles can be extremely useful for the maintenance of the oral mucosa hydration. Adamczak et al. demonstrated the effectiveness of liposomes in water adsorption, desorption, and diffusion, even in environments with high humidity as the oral cavity [279]. To improve the residence time of liposomes in the oral cavity, liposomes can be coated with mucoadhesive polymers like pectin, chitosan, and hydroxyethyl cellulose [279]. The stability of liposomes can be improved by coating them with some of these polymers. This approach can also provide prolonged moisture protection in the oral cavity [280,281]. 

Adamczak et al. also noted that liposomes coated with chitosan and pectin improve their water sorption capacity and mucoadhesion [279]. In another study, Adamczak et al. found that the alginate-coated liposomes have high mucoadhesion without being cytotoxic [280]. Nanostructured systems, such as liposomes, are promising alternatives to promote dry mouth relief because they are composed by phospholipids that bind water in their aqueous compartment [279,280,281] (Table 1). 

Nanoparticles can also be used for preventive purposes, through the use of some compounds to protect the salivary glands from radiation-induced damage and concomitant hyposalivation [278]. 

A summary of the main findings of our review can be found below in Table 1.

## 10. Oral Mucosa Drug Delivery

In recent years, the buccal administration of mucoadhesive formulations has had the greatest interest in the pharmaceutical and dentistry areas. This route of administration is useful due to many advantages, including easy accessibility, patient compliance, and limited enzymatic activity [282]. The administration of drugs in buccal mucosa can also minimize side effects and avoid degradation in the gastrointestinal tract. Another advantage that should be considered is the faster onset of action compared to oral ingestion. One portion of the drug is absorbed through the blood vessels directly to the systemic circulation when administered via the buccal mucosa [282,283].

However, the administration of drugs through the oral mucosa presents some barriers that must be overcome. The short residence time due to the salivary flow may lead to involuntary swallowing. Moreover, only small doses can be administered and drug permeability, controlled drug release, and targeting remain a challenge [282,283]. A promising approach to overcome these challenges is the use of NPs to promote the buccal delivery. NPs are able to improve water solubility and drug dissolution rates, can carry a high drug concentration, and protect drugs from degradation in biological fluids. Furthermore, the ability to improve the mucoadhesive properties of formulations can lead to a prolonged residence time in the oral cavity. 

There are many systems that can promote and improve the buccal administration of drugs. In the past years, a great number of new formulations for this administration route have been developed. Tran et al. classified these systems in three groups: (i) nanoparticle-delivered mucoadhesive films; (ii) nanoparticle-delivered mucoadhesive gels; and (iii) nanoparticle-delivered mucoadhesive solid matrix forms [282]. 

The most commonly used systems are the mucoadhesive films. In these systems, the drug is loaded into nanoparticles and then are incorporated into a mucoadhesive film. Polymeric NPs [284,285,286], liposomes [287,288], chitosan NPs [289,290], lipids and polysaccharides can be used for this aim. These films can also be produced by using hydroxypropyl methyl cellulose (HPMC), carboxymethyl cellulose sodium (SCMC), eudragit, carpobol, and ethyl cellulose. These materials provide mucoadhesion for the formulation and can increase the residence time once in contact with the oral mucosa. 

Castro et al. incorporated PLGA nanoparticles into a mucoadhesive film composed of guar gum [286,291]. This system was developed aiming to allow the buccal application of bioactive peptides that undergo extensive enzymatic degradation. The authors found an increased buccal and intestinal permeation and a significant increase in the mucoadhesive properties [286,291]. 

The buccal mucosa is one of the most promising routes for protein and peptide delivery. There are many studies aiming for the development of non-invasive systems capable to promote the buccal administration of insulin. Insulin is commonly administered through syringes with needles which may lead to low treatment compliance [289]. Al-Nemrawi et al. developed chitosan NPs loaded with insulin, which were dispersed in a film composed by HPMC, SCMC, and carpobol. In vivo studies with diabetic rats showed that the prepared films were able to reduce blood glucose levels when applied buccally [289].

In addition to mucoadhesive films, mucoadhesive gels are also a promising strategy. These gels can be composed of hydroxyethyl cellulose [292], hyaluronic acid-based gel [176], carbopol, and polycarbophil [293,294]. In many cases, the use of nanostructured hydrogels for intraoral administration is better than the commercial products available on the market. Abozaid et al. produced an acyclovir-loaded lipid nanocapsules gel with an enhanced permeation in an ex vivo chicken pouch membrane model compared to a commercial cream [292]. The same results were observed by Muniz et al., who loaded lidocaine and prilocaine into a poly(ε-caprolactone) nanocapsule gel. The developed formulation provided effective and longer-lasting superficial anesthesia in vivo compared to a commercial product [294]. 

Marques et al. developed a mucoadhesive buccal gel containing nanostructured lipid carriers loaded with ibuprofen [293]. This gel presented great residence time on the buccal mucosa and the NLC demonstrated the ability to promote a sustained release of the drug [293]. In the same way, Hosny et al. observed higher ex vivo skin permeability and enhanced antifungal activity with a miconazole self-nanoemulsion in a hydrogel composed of hyaluronic acid [176]. These results demonstrate that it is feasible to use the buccal mucosa for drug administration and it is also possible to reduce the number of daily applications, because these systems present a prolonged drug release. 

Mucoadhesive solid matrix forms are another way to promote the buccal administration of some drugs. Chitosan [295], solid lipids NPs [296], and silymarin NPs [297] can be incorporated in solid matrix to improve mucoadhesion and permeation across the buccal mucosa. Furthermore, solid dosage forms like tablets, sponges, or patches can promote prolonged residence time in the oral cavity when compared to mucoadhesive gels, which can be easily removed by saliva [298].

## 11. Conclusions

The use of NPs is a promising strategy that can help to prevent, shorten treatment duration, or eradicate oral problems such as dental caries, periodontal disease, peri-implantitis, oral candidiasis, and hyposalivation. NPs can also be incorporated into some dental materials, like PMMA and glass ionomer cements, in order to improve the properties of dental prostheses and restorations. Moreover, these particles can help in the early diagnosis of head, neck, and oral cancer, resulting in less morbidity and better prognosis. Further studies, especially clinical trials, still need to be conducted, but these findings indicate that NPs are a step forward towards more efficient dental treatments.

## Figures and Tables

**Table 1:** Summary of the main findings of this review.

Nanoparticle	Association with Other Substances/Materials	Oral Disease/Problem	Potential Applications	Main Findings
CaF_2_NPs	No	Dental caries	Prevention and treatment	Biofilm formation prevention, inhibition of exopolysaccharide production by *Streptococcus mutans*, remineralization of tooth enamel [15].
	Yes—chitosan bioadhesive films			Increase in the residence time of CaF_2_NPs in the oral environment [12].
Chitosan	No	Dental caries	Prevention and treatment	Anti-growth and anti-adherence activity against cariogenic bacteria in vitro [21].
	Yes—sodium fluoride			Slow and continuous fluoride release, with increased release in acidic conditions [13].
	Yes—glass ionomer cement			Increase in the material’s resistance and fluoride release, bactericidal activity [18].
	Yes—glass ionomer cement and titanium oxide (TiO_2_) NPs			Antimicrobial activity with inhibition of biofilm growth, improvement of some physical characteristics [20].
	Yes—copper NPs			Bactericidal activity against *S. mutans* [16].
	Yes—amelogenin-derived peptide QP5			Antibacterial activity and tooth remineralization [24].
PEG-PLGA NPs	Yes—*Dodonaea viscosa* var. *angustifolia* derived flavone	Dental caries	Prevention and treatment	Bactericidal and anti-biofilm effect against *S. mutans* [26].
AgNPs	Yes—azithromycin and clarithromycin	Periodontal disease	Treatment	Antimicrobial efficacy against periodontal disease causing microorganisms [31].
	Yes—electrospun nanofibers			Excellent antibacterial activity [35].
PtNPs	No	Periodontal disease	Treatment	Antibacterial activity against *S. mutans*, *Enterococcus faecalis*, and *Porphyromonas gingivalis* [32].
Bismuth subsalicylate NPs	No	Periodontal disease	Treatment	Antibacterial effect against *Aggregatibacter actinomycetemcomitans, Capnocytophaga gingivalis*, and *P. gingivalis* [33].
PLGA NPs	Yes—BAR peptide	Periodontal disease	Treatment	Disruption of the preformed biofilms more effectively [42].
	Yes—metformin hydrochloride			These NPs decreased inflammation and bone loss [39].
Calcium and zinc-loaded NPs	No	Periodontal disease	Treatment	Periodontal regeneration and precipitation of calcium phosphate deposits [41].
PLA/PLGA NPs	Yes—curcumin	Periodontal disease	Treatment	Inhibition of inflammation and bone resorption [43].
Liposomes	Yes—lidocaine/prilocaine	Periodontal disease	Treatment	This formulation could be a good option to increase patient compliance during treatment [45].
Chitosan	No	Pulp and periapical lesions	Treatment	Chitosan can be used as an intracanal medication [61]; as an effective chelating agent with less alteration in radicular dentine than EDTA [52]; and as as a root canal irrigant with less undesirable effects than NaOCl and chlorhexidine [59].
	Yes—EDTA			This association can simultaneously disinfect root canals and remove the smear layer [98].
AgNPs	No	Pulp and periapical lesions	Treatment	AgNPs are effective against some endodontic-periodontal pathogens [76], and can be used as endodontic irrigants [75].
	Yes—EDTA			EDTA-AgNPs have antimicrobial activity and could be used for effective smear layer removal [83].
Diamond NPs	Yes—gutta percha	Pulp and periapical lesions	Treatment	Decrease in the size of periapical lesions, with no adverse events after 6 months [87].
AuNP and iron oxide NPs	No	Pulp and periapical lesions	Treatment	Inhibition of pathogenic biofilm formation [88,89].
PLGA NPs	Yes—propolis	Pulp and periapical lesions	Treatment	Prolonged release of propolis, enhanced cytocompatibility, and antimicrobial activity [91].
	Yes—moxifloxacin			Sustained antibacterial effect in low doses against *E. faecalis* [6].
AgNPs	No	Peri-implantitis and implant failures	Prevention	AgNPs can be used for the coating of titanium surfaces given their antimicrobial activitiy [120].
TiO_2_NPs	No	Peri-implantitis and implant failures	Prevention	Titanium discs coated with TiO_2_NPs showed significant antibacterial [124] and anticandidal [123] activities.
AuNPs	No	Peri-implantitis and implant failures	Prevention	These NPs showed significantly enhanced osteogenic differentiation and influence on the osseous interface formation [129], and can be used as a bone inductive adjuvant [103].
Chitosan	Yes—AgNPs	Peri-implantitis and implant failures	Prevention	These associations can prevent the surface adhesion of bacteria [132,133].
	Yes—hyaluronic acid			
	Yes—collagen			Improvement of peri-implant tissue attachment [136].
Bismuth NPs	No	Peri-implantitis and implant failures	Treatment	Bismuth-coated titanium surfaces may improve the treatment of peri-implantitis and peri-implant mucositis [122].
Zirconium oxide (ZrO_2_) NPs	Yes—PMMA	Dental prosthesis failures	Prevention	The incorporation of these NPs into PMMA can increase the dimensional accuracy [155] and tensile strength of the denture base acrylic [150], decrease the impact strength [155], and improve the flexural [157] and transverse strength [158].
Silica NPs	Yes—PMMA	Dental prosthesis failures	Prevention	Decrease in the flexural strenght [148,152].
Zinc oxide (ZnO) NPs	Yes—PMMA	Dental prosthesis failures	Prevention	Increase in the hardness, thermal stability, glass transition temperature, and the hydrophilicity of PMMA composites [162,163].
AgNPs	Yes—PMMA	Dental prosthesis failures	Prevention	These NPs affected the transverse strength of the denture base acrylic resins in a concentration-dependent manner and decreased the glass transition temperature [154].
TiO_2_NPs	Yes—PMMA	Dental prosthesis failures	Prevention	TiO_2_NPs can significantly increase the impact strength, tensile strength, and microhardness of acrylic resin [166,167].
Chitosan	No	Oral candidiasis and denture stomatitis	Treatment	Chitosan mouthwash significantly decreased the erythematous area, burning sensation, time required for clinical improvement, and number of blastospores and mycelia [174].
	Yes—curcumin			Chitosan-curcumin mouthwash may serve as a safe and potential topical therapeutic alternative [187].
	Yes—miconazole			Chitosan-based films are a promising approach to deliver miconazole nitrate [189].
	Yes—tissue conditioners			These modified materials present a significant antifungal and antibacterial activity [197,198].
	Yes—denture adhesive			Chitosan has shown properties suitable for the development of an antifungal denture adhesive that could inhibit *C. albicans* adherence [200].
Liposomes	Yes—0WHistatin 5	Oral candidiasis and denture stomatitis	Treatment	This system was able to limit the growth of *C. albicans* [201].
	Yes—doxorubicin	Head, neck, and oral cancer	Treatment	This formulation resulted in a higher percentage of apoptotic cells than doxorubicin alone [239].
Solid lipid NPs	Yes—paclitaxel and ascorbic acid	Head, neck, and oral cancer	Treatment	This combination can be a novel approach for the treatment of oral squamous cell carcinoma [240].
Carbon NPs	No	Head, neck, and oral cancer	Diagnosis	These NPs were able to track occult lingual lymph nodes in early-stage tongue squamous cell carcinoma [258].
AuNPs	No	Head, neck, and oral cancer	Diagnosis	The incorporation of AuNPs have improved the sensitivity of different analyses, such as ELISA [250], optical coherence tomography [251], and surface enhanced Raman spectroscopy [252].
			Treatment	When combined with X-ray irradiation, AuNPs can induce apoptosis [270].
Superparamagnetic iron oxide NPs	Yes—anti-CD44 antibody	Head, neck, and oral cancer	Treatment	Inhibition of tumor growth [264].
Liposomes	No	Hyposalivation	Treatment	Effectiveness in water adsorption, desorption, and diffusion [279].
	Yes—pectin, alginate and chitosan			Increase in the stability [279] and water sorption [281] of liposomes.

## Data Availability

Data sharing not applicable.

## References

[1] Yakop F., Abd Ghafar S.A., Yong Y.K., Saiful Yazan L., Mohamad Hanafiah R., Lim V., Eshak Z. (2018). Silver nanoparticles Clinacanthus Nutans leaves extract induced apoptosis towards oral squamous cell carcinoma cell lines. Artif. Cells Nanomed. Biotechnol..

[2] Rudramurthy G.R., Swamy M.K. (2018). Potential applications of engineered nanoparticles in medicine and biology: An update. J. Biol. Inorg. Chem..

[3] Anu Mary Ealia S., Saravanakumar M.P. (2017). A review on the classification, characterisation, synthesis of nanoparticles and their application. IOP Conf. Ser. Mater. Sci. Eng..

[4] Meena A., Mali H.S., Patnaik A., Kumar S.R. (2017). Comparative investigation of physical, mechanical and thermomechanical characterization of dental composite filled with nanohydroxyapatite and mineral trioxide aggregate. e-Polymers.

[5] Omidi M., Fatehinya A., Farahani M., Akbari Z., Shahmoradi S., Yazdian F., Tahriri M., Moharamzadeh K., Tayebi L., Vashaee D. (2017). Characterization of Biomaterials.

[6] Makkar H., Patri G. (2017). Fabrication and appraisal of poly (Lactic-Co-Glycolic Acid)-moxifloxacin nanoparticles using vitamin E-tpgs: A potential intracanal drug delivery agent. J. Clin. Diagn. Res..

[7] Tanaka M., Okinaga T., Iwanaga K., Matsuo K., Toyono T., Sasaguri M., Ariyoshi W., Tominaga K., Enomoto Y., Matsumura Y. (2019). Anticancer effect of novel platinum nanocomposite beads on oral squamous cell carcinoma cells. J. Biomed. Mater. Res. B Appl. Biomater..

[8] Ketabat F., Pundir M., Mohabatpour F., Lobanova L., Koutsopoulos S., Hadjiiski L., Chen X., Papagerakis P., Papagerakis S. (2019). Controlled drug delivery systems for oral cancer treatment-current status and future perspectives. Pharmaceutics.

[9] Cierech M., Kolenda A., Grudniak A.M., Wojnarowicz J., Wozniak B., Golas M., Swoboda-Kopec E., Lojkowski W., Mierzwinska-Nastalska E. (2016). Significance of polymethylmethacrylate (PMMA) modification by zinc oxide nanoparticles for fungal biofilm formation. Int. J. Pharm..

[10] Selwitz R.H., Ismail A.I., Pitts N.B. (2007). Dental caries. Lancet.

[11] Aida K.L., Kreling P.F., Caiaffa K.S., Calixto G.M.F., Chorilli M., Spolidorio D.M., Santos-Filho N.A., Cilli E.M., Duque C. (2018). Antimicrobial peptide-loaded liquid crystalline precursor bioadhesive system for the prevention of dental caries. Int. J. Nanomed..

[12] Ghafar H., Khan M.I., Sarwar H.S., Yaqoob S., Hussain S.Z., Tariq I., Madni A.U., Shahnaz G., Sohail M.F. (2020). Development and characterization of bioadhesive film embedded with lignocaine and calcium fluoride nanoparticles. AAPS PharmSciTech.

[13] Nguyen S., Escudero C., Sediqi N., Smistad G., Hiorth M. (2017). Fluoride loaded polymeric nanoparticles for dental delivery. Eur. J. Pharm. Sci..

[14] Voltan A.R., Quindós G., Alarcón K.P., Fusco-Almeida A.M., Mendes-Giannini M.J., Chorilli M. (2016). Fungal diseases: Could nanostructured drug delivery systems be a novel paradigm for therapy?. Int. J. Nanomed..

[15] Kulshrestha S., Khan S., Hasan S., Khan M.E., Misba L., Khan A.U. (2016). Calcium fluoride nanoparticles induced suppression of Streptococcus mutans biofilm: An in vitro and in vivo approach. Appl. Microbiol. Biotechnol..

[16] Covarrubias C., Trepiana D., Corral C. (2018). Synthesis of hybrid copper-chitosan nanoparticles with antibacterial activity against cariogenic Streptococcus mutans. Dent. Mater. J..

[17] Samprasit W., Kaomongkolgit R., Sukma M., Rojanarata T., Ngawhirunpat T., Opanasopit P. (2015). Mucoadhesive electrospun chitosan-based nanofibre mats for dental caries prevention. Carbohydr. Polym..

[18] Senthil Kumar R., Ravikumar N., Kavitha S., Mahalaxmi S., Jayasree R., Sampath Kumar T.S., Haneesh M. (2017). Nanochitosan modified glass ionomer cement with enhanced mechanical properties and fluoride release. Int. J. Biol. Macromol..

[19] Ibrahim M.A., Neo J., Esguerra R.J., Fawzy A.S. (2015). Characterization of antibacterial and adhesion properties of chitosan-modified glass ionomer cement. J. Biomater. Appl..

[20] Ibrahim M.A., Meera Priyadarshini B., Neo J., Fawzy A.S. (2017). Characterization of chitosan/TiO(2) nano-powder modified glass-ionomer cement for restorative dental applications. J. Esthet. Restor. Dent..

[21] Aliasghari A., Rabbani Khorasgani M., Vaezifar S., Rahimi F., Younesi H., Khoroushi M. (2016). Evaluation of antibacterial efficiency of chitosan and chitosan nanoparticles on cariogenic streptococci: An in vitro study. Iran. J. Microbiol..

[22] Ashrafi B., Rashidipour M., Marzban A., Soroush S., Azadpour M., Delfani S., Ramak P. (2019). Mentha piperita essential oils loaded in a chitosan nanogel with inhibitory effect on biofilm formation against S. mutans on the dental surface. Carbohydr. Polym..

[23] Ikono R., Vibriani A., Wibowo I., Saputro K.E., Muliawan W., Bachtiar B.M., Mardliyati E., Bachtiar E.W., Rochman N.T., Kagami H. (2019). Nanochitosan antimicrobial activity against Streptococcus mutans and Candida albicans dual-species biofilms. BMC Res. Notes.

[24] Ren Q., Ding L., Li Z., Wang X., Wang K., Han S., Li W., Zhou X., Zhang L. (2019). Chitosan hydrogel containing amelogenin-derived peptide: Inhibition of cariogenic bacteria and promotion of remineralization of initial caries lesions. Arch. Oral Biol..

[25] Liu Y., Busscher H.J., Zhao B., Li Y., Zhang Z., van der Mei H.C., Ren Y., Shi L. (2016). Surface-adaptive, antimicrobially loaded, micellar nanocarriers with enhanced penetration and killing efficiency in staphylococcal biofilms. ACS Nano.

[26] Sebelemetja M., Moeno S., Patel M. (2020). Anti-acidogenic, anti-biofilm and slow release properties of Dodonaea viscosa var. angustifolia flavone stabilized polymeric nanoparticles. Arch. Oral Biol..

[27] Trigo Gutierrez J.K., Zanatta G.C., Ortega A.L.M., Balastegui M.I.C., Sanitá P.V., Pavarina A.C., Barbugli P.A., Mima E.G.O. (2017). Encapsulation of curcumin in polymeric nanoparticles for antimicrobial photodynamic therapy. PLoS ONE.

[28] Zhao Z., Ding C., Wang Y., Tan H., Li J. (2019). pH-Responsive polymeric nanocarriers for efficient killing of cariogenic bacteria in biofilms. Biomater. Sci..

[29] Bao X., Zhao J., Sun J., Hu M., Yang X. (2018). Polydopamine nanoparticles as efficient scavengers for reactive oxygen species in periodontal disease. ACS Nano.

[30] Alvarez Echazú M.I., Olivetti C.E., Peralta I., Alonso M.R., Anesini C., Perez C.J., Alvarez G.S., Desimone M.F. (2018). Development of pH-responsive biopolymer-silica composites loaded with Larrea divaricata Cav. extract with antioxidant activity. Colloids Surf. B Biointerfaces.

[31] Emmanuel R., Palanisamy S., Chen S.M., Chelladurai K., Padmavathy S., Saravanan M., Prakash P., Ajmal Ali M., Al-Hemaid F.M. (2015). Antimicrobial efficacy of green synthesized drug blended silver nanoparticles against dental caries and periodontal disease causing microorganisms. Mater. Sci. Eng. C Mater. Biol. Appl..

[32] Itohiya H., Matsushima Y., Shirakawa S., Kajiyama S., Yashima A., Nagano T., Gomi K. (2019). Organic resolution function and effects of platinum nanoparticles on bacteria and organic matter. PLoS ONE.

[33] Vega-Jiménez A.L., Almaguer-Flores A., Flores-Castañeda M., Camps E., Uribe-Ramírez M., Aztatzi-Aguilar O.G., De Vizcaya-Ruiz A. (2017). Bismuth subsalicylate nanoparticles with anaerobic antibacterial activity for dental applications. Nanotechnology.

[34] Holden M.S., Black J., Lewis A., Boutrin M.C., Walemba E., Sabir T.S., Boskovic D.S., Wilson A., Fletcher H.M., Perry C.C. (2016). Antibacterial activity of partially oxidized Ag/Au nanoparticles against the oral pathogen porphyromonas gingivalis W83. J. Nanomater..

[35] Lee S.J., Heo D.N., Lee D., Heo M., Rim H., Zhang L.G., Park S.A., Do S.H., Moon J.H., Kwon I.K. (2016). One-Step fabrication of AgNPs embedded hybrid dual nanofibrous oral wound dressings. J. Biomed. Nanotechnol..

[36] He Y., Jin Y., Wang X., Yao S., Li Y., Wu Q., Ma G., Cui F., Liu H. (2018). An antimicrobial peptide-loaded gelatin/chitosan nanofibrous membrane fabricated by sequential layer-by-layer electrospinning and electrospraying techniques. Nanomaterials.

[37] Mahmoud M.Y., Sapare S., Curry K.C., Demuth D.R., Steinbach-Rankins J.M. (2019). Rapid release polymeric fibers for inhibition of porphyromonas gingivalis adherence to streptococcus gordonii. Front. Chem..

[38] Backlund C.J., Worley B.V., Sergesketter A.R., Schoenfisch M.H. (2015). Kinetic-dependent Killing of Oral Pathogens with Nitric Oxide. J. Dent. Res..

[39] Pereira A., Brito G.A.C., Lima M.L.S., Silva Júnior A.A.D., Silva E.D.S., de Rezende A.A., Bortolin R.H., Galvan M., Pirih F.Q., Araújo Júnior R.F. (2018). Metformin hydrochloride-loaded PLGA nanoparticle in periodontal disease experimental model using diabetic rats. Int. J. Mol. Sci..

[40] Mou J., Liu Z., Liu J., Lu J., Zhu W., Pei D. (2019). Hydrogel containing minocycline and zinc oxide-loaded serum albumin nanopartical for periodontitis application: Preparation, characterization and evaluation. Drug Deliv..

[41] Osorio R., Alfonso-Rodríguez C.A., Medina-Castillo A.L., Alaminos M., Toledano M. (2016). Bioactive polymeric nanoparticles for periodontal therapy. PLoS ONE.

[42] Kalia P., Jain A., Radha Krishnan R., Demuth D.R., Steinbach-Rankins J.M. (2017). Peptide-modified nanoparticles inhibit formation of Porphyromonas gingivalis biofilms with Streptococcus gordonii. Int. J. Nanomed..

[43] Zambrano L.M.G., Brandao D.A., Rocha F.R.G., Marsiglio R.P., Longo I.B., Primo F.L., Tedesco A.C., Guimaraes-Stabili M.R., Rossa Junior C. (2018). Local administration of curcumin-loaded nanoparticles effectively inhibits inflammation and bone resorption associated with experimental periodontal disease. Sci. Rep..

[44] Wijetunge S.S., Wen J., Yeh C.K., Sun Y. (2020). Wheat germ agglutinin liposomes with surface grafted cyclodextrins as bioadhesive dual-drug delivery nanocarriers to treat oral cells. Colloids Surf. B Biointerfaces.

[45] Moraes G.S., Santos I.B.D., Pinto S.C.S., Pochapski M.T., Farago P.V., Pilatti G.L., Santos F.A. (2020). Liposomal anesthetic gel for pain control during periodontal therapy in adults: A placebo-controlled RCT. J. Appl. Oral Sci..

[46] Klein-Júnior C.A., Reston E., Plepis A.M., Martins V.C., Pötter I.C., Lundy F., Hentschke G.S., Hentschke V.S., Karim I.E. (2019). Development and evaluation of calcium hydroxide-coated, pericardium-based biomembranes for direct pulp capping. J. Investig. Clin. Dent..

[47] Elshinawy M.I., Al-Madboly L.A., Ghoneim W.M., El-Deeb N.M. (2018). Synergistic effect of newly introduced root canal medicaments; ozonated olive oil and chitosan nanoparticles, against persistent endodontic pathogens. Front. Microbiol..

[48] Nair N., James B., Devadathan A., Johny M.K., Mathew J., Jacob J. (2018). Comparative evaluation of antibiofilm efficacy of chitosan nanoparticle- and zinc oxide nanoparticle-incorporated calcium hydroxide-based sealer: An in vitro study. Contemp. Clin. Dent..

[49] Teixeira A.B.V., de Castro D.T., Schiavon M.A., Dos Reis A.C. (2020). Cytotoxicity and release ions of endodontic sealers incorporated with a silver and vanadium base nanomaterial. Odontology.

[50] Afkhami F., Elahy S., Mahmoudi-Nahavandi A. (2017). Spectrophotometric analysis of crown discoloration following the use of silver nanoparticles combined with calcium hydroxide as intracanal medicament. J. Clin. Exp. Dent..

[51] Antunes P.V.S., Flamini L.E.S., Chaves J.F.M., Silva R.G., Cruz Filho A.M.D. (2020). Comparative effects of final canal irrigation with chitosan and EDTA. J. Appl. Oral Sci..

[52] Mathew S.P., Pai V.S., Usha G., Nadig R.R. (2017). Comparative evaluation of smear layer removal by chitosan and ethylenediaminetetraacetic acid when used as irrigant and its effect on root dentine: An in vitro atomic force microscopic and energy-dispersive X-ray analysis. J. Conserv. Dent..

[53] Kesim B., Burak A.K., Ustun Y., Delikan E., Gungor A. (2018). Effect of chitosan on sealer penetration into the dentinal tubules. Niger. J. Clin. Pract..

[54] Pivatto K., Pedro F.L.M., Guedes O.A., Silva A.F.D., Piva E., Pereira T.M., Rosa W., Borges A.H. (2020). Cytotoxicity of chelating agents used in endodontics and their influence on MMPs of cell membranes. Braz. Dent. J..

[55] Raghu R., Pradeep G., Shetty A., Gautham P.M., Puneetha P.G., Reddy T.V.S. (2017). Retrievability of calcium hydroxide intracanal medicament with three calcium chelators, ethylenediaminetetraacetic acid, citric acid, and chitosan from root canals: An in vitro cone beam computed tomography volumetric analysis. J. Conserv. Dent..

[56] El Ashiry E.A., Alamoudi N.M., El Ashiry M.K., Bastawy H.A., El Derwi D.A., Atta H.M. (2018). Tissue engineering of necrotic dental pulp of immature teeth with apical periodontitis in dogs: Radiographic and histological evaluation. J. Clin. Pediatr. Dent..

[57] Ji Y., Choi S.K., Sultan A.S., Chuncai K., Lin X., Dashtimoghadam E., Melo M.A., Weir M., Xu H., Tayebi L. (2018). Nanomagnetic-mediated drug delivery for the treatment of dental disease. Nanomedicine.

[58] Uğur Aydin Z., Akpinar K.E., Hepokur C., Erdönmez D. (2018). Assessment of toxicity and oxidative DNA damage of sodium hypochlorite, chitosan and propolis on fibroblast cells. Braz. Oral Res..

[59] Yadav P., Chaudhary S., Saxena R.K., Talwar S., Yadav S. (2017). Evaluation of antimicrobial and antifungal efficacy of chitosan as endodontic irrigant against enterococcus faecalis and candida albicans biofilm formed on tooth substrate. J. Clin. Exp. Dent..

[60] Kamble A.B., Abraham S., Kakde D.D., Shashidhar C., Mehta D.L. (2017). Scanning electron microscopic evaluation of efficacy of 17% ethylenediaminetetraacetic acid and chitosan for smear layer removal with ultrasonics: An in vitro study. Contemp. Clin. Dent..

[61] Sireesha A., Jayasree R., Vidhya S., Mahalaxmi S., Sujatha V., Kumar T.S.S. (2017). Comparative evaluation of micron- and nano-sized intracanal medicaments on penetration and fracture resistance of root dentin-An in vitro study. Int. J. Biol. Macromol..

[62] Del Carpio-Perochena A., Kishen A., Felitti R., Bhagirath A.Y., Medapati M.R., Lai C., Cunha R.S. (2017). Antibacterial roperties of chitosan nanoparticles and propolis associated with calcium hydroxide against single- and multispecies biofilms: An in vitro and in situ study. J. Endod..

[63] Farhadian N., Godiny M., Moradi S., Hemati Azandaryani A., Shahlaei M. (2018). Chitosan/gelatin as a new nano-carrier system for calcium hydroxide delivery in endodontic applications: Development, characterization and process optimization. Mater. Sci. Eng. C Mater. Biol. Appl..

[64] Thota M.M., Sudha K., Malini D.L., Madhavi S.B. (2017). Effect of different irrigating solutions on depth of penetration of sealer into dentinal tubules: A confocal microscopic study. Contemp. Clin. Dent..

[65] Ozlek E., Rath P.P., Kishen A., Neelakantan P. (2020). A chitosan-based irrigant improves the dislocation resistance of a mineral trioxide aggregate-resin hybrid root canal sealer. Clin. Oral Investig..

[66] Paiola F.G., Lopes F.C., Mazzi-Chaves J.F., Pereira R.D., Oliveira H.F., Queiroz A.M., Sousa-Neto M.D. (2018). How to improve root canal filling in teeth subjected to radiation therapy for cancer. Braz. Oral Res..

[67] Flores-Arriaga J.C., Pozos-Guillén A.J., González-Ortega O., Escobar-García D.M., Masuoka-Ito D., Del Campo-Téllez B.I.M., Cerda-Cristerna B.I. (2019). Calcium sustained release, pH changes and cell viability induced by chitosan-based pastes for apexification. Odontology.

[68] Savitha A., SriRekha A., Vijay R., Ashwija, Champa C., Jaykumar T. (2019). An in vivo comparative evaluation of antimicrobial efficacy of chitosan, chlorhexidine gluconate gel and their combination as an intracanal medicament against Enterococcus faecalis in failed endodontic cases using real time polymerase chain reaction (qPCR). Saudi Dent. J..

[69] Zhu N., Chatzistavrou X., Ge L., Qin M., Papagerakis P., Wang Y. (2019). Biological properties of modified bioactive glass on dental pulp cells. J. Dent..

[70] Wu S., Zhou Y., Yu Y., Zhou X., Du W., Wan M., Fan Y., Zhou X., Xu X., Zheng L. (2019). Evaluation of chitosan hydrogel for sustained delivery of VEGF for odontogenic differentiation of dental pulp stem cells. Stem Cells Int..

[71] Bordini E.A.F., Cassiano F.B., Silva I.S.P., Usberti F.R., Anovazzi G., Pacheco L.E., Pansani T.N., Leite M.L., Hebling J., de Souza Costa C.A. (2020). Synergistic potential of 1α,25-dihydroxyvitamin D3 and calcium-aluminate-chitosan scaffolds with dental pulp cells. Clin. Oral Investig..

[72] Balata G.F., Abdelhady M.I.S., Mahmoud G.M., Matar M.A., Abd El-Latif A.N. (2018). Formulation of saudi propolis into biodegradable chitosan chips for vital pulpotomy. Curr. Drug Deliv..

[73] Mittal N., Parashar V. (2019). Regenerative evaluation of immature roots using PRF and artificial scaffolds in necrotic permanent teeth: A clinical study. J. Contemp. Dent. Pract..

[74] Palma P.J., Ramos J.C., Martins J.B., Diogenes A., Figueiredo M.H., Ferreira P., Viegas C., Santos J.M. (2017). Histologic evaluation of regenerative endodontic procedures with the Use of chitosan scaffolds in immature dog teeth with apical periodontitis. J. Endod..

[75] de Almeida J., Cechella B.C., Bernardi A.V., de Lima Pimenta A., Felippe W.T. (2018). Effectiveness of nanoparticles solutions and conventional endodontic irrigants against enterococcus faecalis biofilm. Indian J. Dent. Res..

[76] Halkai K.R., Halkai R., Mudda J.A., Shivanna V., Rathod V. (2018). Antibiofilm efficacy of biosynthesized silver nanoparticles against endodontic-periodontal pathogens: An in vitro study. J. Conserv. Dent..

[77] Zheng T., Huang X., Chen J., Feng D., Mei L., Huang Y., Quan G., Zhu C., Singh V., Ran H. (2018). A liquid crystalline precursor incorporating chlorhexidine acetate and silver nanoparticles for root canal disinfection. Biomater. Sci..

[78] Sadek R.W., Moussa S.M., El Backly R.M., Hammouda A.F. (2019). Evaluation of the efficacy of three antimicrobial agents used for regenerative endodontics: An in vitro study. Microb. Drug Resist..

[79] Halkai K.R., Mudda J.A., Shivanna V., Rathod V., Halkai R. (2018). Antibacterial efficacy of biosynthesized silver nanoparticles against enterococcus faecalis biofilm: An in vitro study. Contemp. Clin. Dent..

[80] Takamiya A.S., Monteiro D.R., Bernabé D.G., Gorup L.F., Camargo E.R., Gomes-Filho J.E., Oliveira S.H., Barbosa D.B. (2016). In vitro and in vivo toxicity evaluation of colloidal silver nanoparticles used in endodontic treatments. J. Endod..

[81] Chávez-Andrade G.M., Tanomaru-Filho M., Rodrigues E.M., Gomes-Cornélio A.L., Faria G., Bernardi M.I.B., Guerreiro-Tanomaru J.M. (2017). Cytotoxicity, genotoxicity and antibacterial activity of poly(vinyl alcohol)-coated silver nanoparticles and farnesol as irrigating solutions. Arch. Oral Biol..

[82] Ioannidis K., Niazi S., Mylonas P., Mannocci F., Deb S. (2019). The synthesis of nano silver-graphene oxide system and its efficacy against endodontic biofilms using a novel tooth model. Dent. Mater..

[83] Martinez-Andrade J.M., Avalos-Borja M., Vilchis-Nestor A.R., Sanchez-Vargas L.O., Castro-Longoria E. (2018). Dual function of EDTA with silver nanoparticles for root canal treatment-A novel modification. PLoS ONE.

[84] Baras B.H., Sun J., Melo M.A.S., Tay F.R., Oates T.W., Zhang K., Weir M.D., Xu H.H.K. (2019). Novel root canal sealer with dimethylaminohexadecyl methacrylate, nano-silver and nano-calcium phosphate to kill bacteria inside root dentin and increase dentin hardness. Dent. Mater..

[85] Chávez-Andrade G.M., Tanomaru-Filho M., Basso Bernardi M.I., de Toledo Leonardo R., Faria G., Guerreiro-Tanomaru J.M. (2019). Antimicrobial and biofilm anti-adhesion activities of silver nanoparticles and farnesol against endodontic microorganisms for possible application in root canal treatment. Arch. Oral Biol..

[86] Moazami F., Sahebi S., Ahzan S. (2018). Tooth discoloration induced by imidazolium based silver nanoparticles as an intracanal irrigant. J. Dent..

[87] Lee D.K., Kee T., Liang Z., Hsiou D., Miya D., Wu B., Osawa E., Chow E.K., Sung E.C., Kang M.K. (2017). Clinical validation of a nanodiamond-embedded thermoplastic biomaterial. Proc. Natl. Acad. Sci. USA.

[88] Yu Q., Li J., Zhang Y., Wang Y., Liu L., Li M. (2016). Inhibition of gold nanoparticles (AuNPs) on pathogenic biofilm formation and invasion to host cells. Sci. Rep..

[89] Bukhari S., Kim D., Liu Y., Karabucak B., Koo H. (2018). Novel endodontic disinfection approach using catalytic nanoparticles. J. Endod..

[90] Wang L., Xie X., Li C., Liu H., Zhang K., Zhou Y., Chang X., Xu H.H.K. (2017). Novel bioactive root canal sealer to inhibit endodontic multispecies biofilms with remineralizing calcium phosphate ions. J. Dent..

[91] Abdel Raheem I.A., Abdul Razek A., Elgendy A.A., Saleh N.M., Shaaban M.I., Abd El-Hady F.K. (2019). Design, evaluation and antimicrobial activity of egyptian propolis-loaded nanoparticles: Intrinsic role as a novel and naturally based root canal nanosealer. Int. J. Nanomed..

[92] Abdelmonem R., Younis M.K., Hassan D.H., El-Sayed Ahmed M.A.E., Hassanein E., El-Batouty K., Elfaham A. (2019). Formulation and characterization of chlorhexidine HCl nanoemulsion as a promising antibacterial root canal irrigant: In-vitro and ex-vivo studies. Int. J. Nanomed..

[93] Imura K., Hashimoto Y., Okada M., Yoshikawa K., Yamamoto K. (2019). Application of hydroxyapatite nanoparticle-assembled powder using basic fibroblast growth factor as a pulp-capping agent. Dent. Mater. J..

[94] Afkhami F., Akbari S., Chiniforush N. (2017). Entrococcus faecalis elimination in root canals using silver nanoparticles, Photodynamic therapy, diode laser, or laser-activated nanoparticles: An in vitro study. J. Endod..

[95] Camacho-Alonso F., Julián-Belmonte E., Chiva-García F., Martínez-Beneyto Y. (2017). Bactericidal efficacy of photodynamic therapy and chitosan in root canals experimentally infected with enterococcus faecalis: An in vitro study. Photomed. Laser Surg..

[96] Kushwaha V., Yadav R.K., Tikku A.P., Chandra A., Verma P., Gupta P., Shakya V.K. (2018). Comparative evaluation of antibacterial effect of nanoparticles and lasers against endodontic microbiota: An in vitro study. J. Clin. Exp. Dent..

[97] Abraham S., Vaswani S.D., Najan H.B., Mehta D.L., Kamble A.B., Chaudhari S.D. (2019). Scanning electron microscopic evaluation of smear layer removal at the apical third of root canals using diode laser, endoActivator, and ultrasonics with chitosan: An in vitro study. J. Conserv. Dent..

[98] Geethapriya N., Subbiya A., Padmavathy K., Mahalakshmi K., Vivekanandan P., Sukumaran V.G. (2016). Effect of chitosan-ethylenediamine tetraacetic acid on Enterococcus faecalis dentinal biofilm and smear layer removal. J. Conserv. Dent..

[99] Glenn B., Drum M., Reader A., Fowler S., Nusstein J., Beck M. (2016). Does liposomal bupivacaine (exparel) significantly reduce postoperative pain/numbness in symptomatic teeth with a diagnosis of necrosis? A prospective, randomized, double-blind trial. J. Endod..

[100] Bultema K., Fowler S., Drum M., Reader A., Nusstein J., Beck M. (2016). Pain reduction in untreated symptomatic irreversible pulpitis using liposomal bupivacaine (exparel): A prospective, randomized, double-blind trial. J. Endod..

[101] Al-Hashedi A.A., Laurenti M., Amine Mezour M., Basiri T., Touazine H., Jahazi M., Tamimi F. (2019). Advanced inorganic nanocomposite for decontaminating titanium dental implants. J. Biomed. Mater. Res. B Appl. Biomater..

[102] Fröber K., Bergs C., Pich A., Conrads G. (2020). Biofunctionalized zinc peroxide nanoparticles inhibit peri-implantitis associated anaerobes and Aggregatibacter actinomycetemcomitans pH-dependent. Anaerobe.

[103] Jadhav K., Hr R., Deshpande S., Jagwani S., Dhamecha D., Jalalpure S., Subburayan K., Baheti D. (2018). Phytosynthesis of gold nanoparticles: Characterization, biocompatibility, and evaluation of its osteoinductive potential for application in implant dentistry. Mater. Sci. Eng. C Mater. Biol. Appl..

[104] Govindharajulu J.P., Chen X., Li Y., Rodriguez-Cabello J.C., Battacharya M., Aparicio C. (2017). Chitosan-recombinamer layer-by-layer coatings for multifunctional implants. Int. J. Mol. Sci..

[105] Divakar D.D., Jastaniyah N.T., Altamimi H.G., Alnakhli Y.O., Muzaheed, Alkheraif A.A., Haleem S. (2018). Enhanced antimicrobial activity of naturally derived bioactive molecule chitosan conjugated silver nanoparticle against dental implant pathogens. Int. J. Biol. Macromol..

[106] Jin J., Zhang L., Shi M., Zhang Y., Wang Q. (2017). Ti-GO-Ag nanocomposite: The effect of content level on the antimicrobial activity and cytotoxicity. Int. J. Nanomed..

[107] Larsen O.I., Enersen M., Kristoffersen A.K., Wennerberg A., Bunaes D.F., Lie S.A., Leknes K.N. (2017). Antimicrobial effects of three different treatment modalities on dental implant surfaces. J. Oral Implantol..

[108] Li W., Yang Y., Zhang H., Xu Z., Zhao L., Wang J., Qiu Y., Liu B. (2019). Improvements on biological and antimicrobial properties of titanium modified by AgNPs-loaded chitosan-heparin polyelectrolyte multilayers. J. Mater. Sci. Mater. Med..

[109] Valverde A., Perez-Alvarez L., Ruiz-Rubio L., Pacha Olivenza M.A., Garcia Blanco M.B., Diaz-Fuentes M., Vilas-Vilela J.L. (2019). Antibacterial hyaluronic acid/chitosan multilayers onto smooth and micropatterned titanium surfaces. Carbohydr. Polym..

[110] Roguska A., Belcarz A., Zalewska J., Holdynski M., Andrzejczuk M., Pisarek M., Ginalska G. (2018). Metal TiO2 nanotube layers for the treatment of dental implant infections. ACS Appl. Mater. Interfaces.

[111] Rosenbaum J., Versace D.L., Abbad-Andallousi S., Pires R., Azevedo C., Cenedese P., Dubot P. (2017). Antibacterial properties of nanostructured Cu-TiO2 surfaces for dental implants. Biomater. Sci..

[112] Sobolev A., Valkov A., Kossenko A., Wolicki I., Zinigrad M., Borodianskiy K. (2019). Bioactive coating on Ti alloy with high osseointegration and antibacterial Ag nanoparticles. ACS Appl. Mater. Interfaces.

[113] Venugopal A., Muthuchamy N., Tejani H., Gopalan A.I., Lee K.P., Lee H.J., Kyung H.M. (2017). Incorporation of silver nanoparticles on the surface of orthodontic microimplants to achieve antimicrobial properties. Korean J. Orthod..

[114] Wohlfahrt J.C., Aass A.M., Koldsland O.C. (2019). Treatment of peri-implant mucositis with a chitosan brush-A pilot randomized clinical trial. Int. J. Dent. Hyg..

[115] Wohlfahrt J.C., Evensen B.J., Zeza B., Jansson H., Pilloni A., Roos-Jansaker A.M., Di Tanna G.L., Aass A.M., Klepp M., Koldsland O.C. (2017). A novel non-surgical method for mild peri-implantitis- a multicenter consecutive case series. Int. J. Implant. Dent..

[116] Cao X., Cai X., Chen R., Zhang H., Jiang T., Wang Y. (2019). A thermosensitive chitosan-based hydrogel for sealing and lubricating purposes in dental implant system. Clin. Implant. Dent. Relat. Res..

[117] De Leo V., Mattioli-Belmonte M., Cimmarusti M.T., Panniello A., Dicarlo M., Milano F., Agostiano A., De Giglio E., Catucci L. (2017). Liposome-modified titanium surface: A strategy to locally deliver bioactive molecules. Colloids Surf. B Biointerfaces.

[118] Song J., Chen Q., Zhang Y., Diba M., Kolwijck E., Shao J., Jansen J.A., Yang F., Boccaccini A.R., Leeuwenburgh S.C. (2016). Electrophoretic deposition of chitosan coatings modified with gelatin nanospheres to tune the release of antibiotics. ACS Appl. Mater. Interfaces.

[119] Pokrowiecki R., Zaręba T., Szaraniec B., Pałka K., Mielczarek A., Menaszek E., Tyski S. (2017). In vitro studies of nanosilver-doped titanium implants for oral and maxillofacial surgery. Int. J. Nanomed..

[120] Lampé I., Beke D., Biri S., Csarnovics I., Csik A., Dombrádi Z., Hajdu P., Hegedűs V., Rácz R., Varga I. (2019). Investigation of silver nanoparticles on titanium surface created by ion implantation technology. Int. J. Nanomed..

[121] Choi S.H., Jang Y.S., Jang J.H., Bae T.S., Lee S.J., Lee M.H. (2019). Enhanced antibacterial activity of titanium by surface modification with polydopamine and silver for dental implant application. J. Appl. Biomater. Funct. Mater..

[122] Gosau M., Haupt M., Thude S., Strowitzki M., Schminke B., Buergers R. (2016). Antimicrobial effect and biocompatibility of novel metallic nanocrystalline implant coatings. J. Biomed. Mater. Res. B Appl. Biomater..

[123] Chidambaranathan A.S., Mohandoss K., Balasubramaniam M.K. (2016). Comparative evaluation of antifungal effect of titanium, Zirconium and Aluminium nanoparticles coated Titanium plates against C. albicans. J. Clin. Diagn. Res..

[124] Karthikeyan M., Ahila S.C., Muthu Kumar B. (2019). The antibacterial influence of nanotopographic titanium, zirconium, and aluminum nanoparticles against Staphylococcus aureus and porphyromonas gingivalis: An in vitro study. Indian J. Dent. Res..

[125] Salaie R.N., Besinis A., Le H., Tredwin C., Handy R.D. (2020). The biocompatibility of silver and nanohydroxyapatite coatings on titanium dental implants with human primary osteoblast cells. Mater. Sci. Eng. C Mater. Biol. Appl..

[126] Martinez E.F., Ishikawa G.J., de Lemos A.B., Barbosa Bezerra F.J., Sperandio M., Napimoga M.H. (2018). Evaluation of a Titanium surface treated with hydroxyapatite nanocrystals on osteoblastic cell behavior: An in vitro study. Int. J. Oral Maxillofac. Implants.

[127] de Lima Cavalcanti J.H., Matos P.C., Depes de Gouvea C.V., Carvalho W., Calvo-Guirado J.L., Aragoneses J.M., Perez-Diaz L., Gehrke S.A. (2019). In vitro assessment of the functional dynamics of titanium with surface coating of hydroxyapatite Nanoparticles. Materials.

[128] Suo L., Jiang N., Wang Y., Wang P., Chen J., Pei X., Wang J., Wan Q. (2019). The enhancement of osseointegration using a graphene oxide/chitosan/hydroxyapatite composite coating on titanium fabricated by electrophoretic deposition. J. Biomed. Mater. Res. B Appl. Biomater..

[129] Heo D.N., Ko W.K., Lee H.R., Lee S.J., Lee D., Um S.H., Lee J.H., Woo Y.H., Zhang L.G., Lee D.W. (2016). Titanium dental implants surface-immobilized with gold nanoparticles as osteoinductive agents for rapid osseointegration. J. Colloid Interface Sci..

[130] Kalyoncuoglu U.T., Yilmaz B., Koc S.G., Evis Z., Arpaci P.U., Kansu G. (2018). Investigation of surface structure and biocompatibility of chitosan-coated zirconia and alumina dental abutments. Clin. Implant Dent. Relat. Res..

[131] Zhong X., Song Y., Yang P., Wang Y., Jiang S., Zhang X., Li C. (2016). Titanium surface priming with phase-transited lysozyme to establish a silver nanoparticle-loaded chitosan/hyaluronic acid antibacterial multilayer via layer-by-layer self-assembly. PLoS ONE.

[132] Cheng Y.F., Zhang J.Y., Wang Y.B., Li C.M., Lu Z.S., Hu X.F., Xu L.Q. (2019). Deposition of catechol-functionalized chitosan and silver nanoparticles on biomedical titanium surfaces for antibacterial application. Mater. Sci. Eng. C Mater. Biol. Appl..

[133] Guan B., Wang H., Xu R., Zheng G., Yang J., Liu Z., Cao M., Wu M., Song J., Li N. (2016). Establishing antibacterial multilayer films on the surface of direct metal laser sintered Titanium primed with phase-transited lysozyme. Sci. Rep..

[134] Yuan Z., Huang S., Lan S., Xiong H., Tao B., Ding Y., Liu Y., Liu P., Cai K. (2018). Surface engineering of titanium implants with enzyme-triggered antibacterial properties and enhanced osseointegration in vivo. J. Mater. Chem. B.

[135] Yang M., Jiang P., Ge Y., Lan F., Zhou X., He J., Wu Y. (2018). Dopamine self-polymerized along with hydroxyapatite onto the preactivated titanium percutaneous implants surface to promote human gingival fibroblast behavior and antimicrobial activity for biological sealing. J. Biomater. Appl..

[136] Wang J., He X.T., Xu X.Y., Yin Y., Li X., Bi C.S., Hong Y.L., Chen F.M. (2019). Surface modification via plasmid-mediated pLAMA3-CM gene transfection promotes the attachment of gingival epithelial cells to titanium sheets in vitro and improves biological sealing at the transmucosal sites of titanium implants in vivo. J. Mater. Chem. B.

[137] Palla-Rubio B., Araújo-Gomes N., Fernández-Gutiérrez M., Rojo L., Suay J., Gurruchaga M., Goñi I. (2019). Synthesis and characterization of silica-chitosan hybrid materials as antibacterial coatings for titanium implants. Carbohydr. Polym..

[138] Bonifacio M.A., Cometa S., Dicarlo M., Baruzzi F., de Candia S., Gloria A., Giangregorio M.M., Mattioli-Belmonte M., De Giglio E. (2017). Gallium-modified chitosan/poly(acrylic acid) bilayer coatings for improved titanium implant performances. Carbohydr. Polym..

[139] Belouka S.M., Strietzel F.P. (2016). Sinus floor elevation and augmentation using synthetic nanocrystalline and nanoporous hydroxyapatite bone substitute materials: Preliminary histologic results. Int. J. Oral Maxillofac. Implants.

[140] Khaled H., Atef M., Hakam M. (2019). Maxillary sinus floor elevation using hydroxyapatite nano particles vs tenting technique with simultaneous implant placement: A randomized clinical trial. Clin. Implant Dent. Relat. Res..

[141] Lee Y.H., Kim J.S., Kim J.E., Lee M.H., Jeon J.G., Park I.S., Yi H.K. (2017). Nanoparticle mediated PPARgamma gene delivery on dental implants improves osseointegration via mitochondrial biogenesis in diabetes mellitus rat model. Nanomedicine.

[142] Takanche J.S., Kim J.E., Kim J.S., Lee M.H., Jeon J.G., Park I.S., Yi H.K. (2018). Chitosan-gold nanoparticles mediated gene delivery of c-myb facilitates osseointegration of dental implants in ovariectomized rat. Artif. Cells Nanomed. Biotechnol..

[143] Xing H., Wang X., Xiao S., Zhang G., Li M., Wang P., Shi Q., Qiao P., E L., Liu H. (2017). Osseointegration of layer-by-layer polyelectrolyte multilayers loaded with IGF1 and coated on titanium implant under osteoporotic condition. Int. J. Nanomed..

[144] Iero P.T., Mulherin D.R., Jensen O., Berry T., Danesi H., Razook S.J. (2018). A prospective, randomized, open-label study comparing an opioid-sparing postsurgical pain management protocol with and without liposomal bupivacaine for full-arch implant surgery. Int. J. Oral Maxillofac. Implant..

[145] Totu E.E., Nechifor A.C., Nechifor G., Aboul-Enein H.Y., Cristache C.M. (2017). Poly(methyl methacrylate) with TiO2 nanoparticles inclusion for stereolitographic complete denture manufacturing-the fututre in dental care for elderly edentulous patients?. J. Dent..

[146] Carlsson G.E., Omar R. (2010). The future of complete dentures in oral rehabilitation. A critical review. J. Oral Rehabil..

[147] Al-Harbi F.A., Abdel-Halim M.S., Gad M.M., Fouda S.M., Baba N.Z., AlRumaih H.S., Akhtar S. (2019). Effect of nanodiamond addition on flexural strength, impact strength, and surface roughness of PMMA denture base. J. Prosthodont..

[148] Cevik P., Yildirim-Bicer A.Z. (2018). The effect of silica and prepolymer nanoparticles on the mechanical properties of denture base acrylic resin. J. Prosthodont..

[149] Gad M.M., Al-Thobity A.M., Rahoma A., Abualsaud R., Al-Harbi F.A., Akhtar S. (2019). Reinforcement of PMMA denture base material with a mixture of ZrO2 nanoparticles and glass fibers. Int. J. Dent..

[150] Gad M.M., Abualsaud R., Rahoma A., Al-Thobity A.M., Al-Abidi K.S., Akhtar S. (2018). Effect of zirconium oxide nanoparticles addition on the optical and tensile properties of polymethyl methacrylate denture base material. Int. J. Nanomed..

[151] Oyar P., Sana F.A., Nasseri B., Durkan R. (2018). Effect of green gold nanoparticles synthesized with plant on the flexural strength of heat-polymerized acrylic resin. Niger. J. Clin. Pract..

[152] Karci M., Demir N., Yazman S. (2019). Evaluation of flexural strength of different denture base materials reinforced with different nanoparticles. J. Prosthodont..

[153] Chen R., Han Z., Huang Z., Karki J., Wang C., Zhu B., Zhang X. (2017). Antibacterial activity, cytotoxicity and mechanical behavior of nano-enhanced denture base resin with different kinds of inorganic antibacterial agents. Dent. Mater. J..

[154] Koroglu A., Sahin O., Kurkcuoglu I., Dede D.O., Ozdemir T., Hazer B. (2016). Silver nanoparticle incorporation effect on mechanical and thermal properties of denture base acrylic resins. J. Appl. Oral Sci..

[155] Begum S.S., Ajay R., Devaki V., Divya K., Balu K., Kumar P.A. (2019). Impact strength and dimensional accuracy of heat-cure denture base resin reinforced with ZrO2 nanoparticles: An in vitro study. J. Pharm. Bioallied Sci..

[156] Yoshizaki T., Akiba N., Inokoshi M., Shimada M., Minakuchi S. (2017). Hydrophilic nano-silica coating agents with platinum and diamond nanoparticles for denture base materials. Dent. Mater. J.

[157] Gad M.M., Rahoma A., Al-Thobity A.M., ArRejaie A.S. (2016). Influence of incorporation of ZrO2 nanoparticles on the repair strength of polymethyl methacrylate denture bases. Int. J. Nanomed..

[158] Gad M., ArRejaie A.S., Abdel-Halim M.S., Rahoma A. (2016). The reinforcement effect of nano-zirconia on the transverse strength of repaired acrylic denture base. Int. J. Dent..

[159] Gad M.M., Al-Thobity A.M., Shahin S.Y., Alsaqer B.T., Ali A.A. (2017). Inhibitory effect of zirconium oxide nanoparticles on Candida albicans adhesion to repaired polymethyl methacrylate denture bases and interim removable prostheses: A new approach for denture stomatitis prevention. Int. J. Nanomed..

[160] Jiangkongkho P., Arksornnukit M., Takahashi H. (2018). The synthesis, modification, and application of nanosilica in polymethyl methacrylate denture base. Dent. Mater. J.

[161] Anaraki M.R., Jangjoo A., Alimoradi F., Maleki Dizaj S., Lotfipour F. (2017). Comparison of antifungal properties of acrylic resin reinforced with ZnO and Ag nanoparticles. Pharm. Sci..

[162] Cierech M., Osica I., Kolenda A., Wojnarowicz J., Szmigiel D., Lojkowski W., Kurzydlowski K., Ariga K., Mierzwinska-Nastalska E. (2018). Mechanical and physicochemical properties of newly formed ZnO-PMMA nanocomposites for denture bases. Nanomaterials.

[163] Salahuddin N., El-Kemary M., Ibrahim E. (2017). Reinforcement of polymethyl methacrylate denture base resin with ZnO nanostructures. Int. J. Appl. Ceram. Technol..

[164] Anwander M., Rosentritt M., Schneider-Feyrer S., Hahnel S. (2017). Biofilm formation on denture base resin including ZnO, CaO, and TiO2 nanoparticles. J. Adv. Prosthodont..

[165] Kamonkhantikul K., Arksornnukit M., Takahashi H. (2017). Antifungal, optical, and mechanical properties of polymethylmethacrylate material incorporated with silanized zinc oxide nanoparticles. Int. J. Nanomed..

[166] Ashour M., El-Shennawy M., Althomali Y., Omar A. (2016). Effect of Titanium dioxide nano particles incorporation on mechanical and physical properties on two different types of acrylic resin denture base. World J. Nano Sci. Eng..

[167] Ghahremani L., Shirkavand S., Akbari F., Sabzikari N. (2017). Tensile strength and impact strength of color modified acrylic resin reinforced with titanium dioxide nanoparticles. J. Clin. Exp. Dent..

[168] Ghahremanloo A., Movahedzadeh M. (2016). The effect of silver nano particles on Candida albicans and streptococcus mutans in denture acrylic resins. J. Dent. Mater. Tech..

[169] Li Z., Sun J., Lan J., Qi Q. (2016). Effect of a denture base acrylic resin containing silver nanoparticles on Candida albicans adhesion and biofilm formation. Gerodontology.

[170] de Castro D.T., Valente M.L., Agnelli J.A., Lovato da Silva C.H., Watanabe E., Siqueira R.L., Alves O.L., Holtz R.D., dos Reis A.C. (2016). In vitro study of the antibacterial properties and impact strength of dental acrylic resins modified with a nanomaterial. J. Prosthet. Dent..

[171] Sadeghi Ardestani Z., Falahati M., Sayah Alborzi S., Ashrafi Khozani M., Rostam Khani F., Bahador A. (2016). The effect of nanochitosans particles on Candida biofilm formation. Curr. Med. Mycol..

[172] Bongomin F., Gago S., Oladele R.O., Denning D.W. (2017). Global and multi-national prevalence of fungal diseases-estimate precision. J. Fungi.

[173] Pinto Reis C., Vasques Roque L., Baptista M., Rijo P. (2016). Innovative formulation of nystatin particulate systems in toothpaste for candidiasis treatment. Pharm. Dev. Technol..

[174] Atai Z., Atai M., Amini J., Salehi N. (2017). In vivo study of antifungal effects of low-molecular-weight chitosan against Candida albicans. J. Oral Sci..

[175] Mima E.G., Vergani C.E., Machado A.L., Massucato E.M., Colombo A.L., Bagnato V.S., Pavarina A.C. (2012). Comparison of Photodynamic Therapy versus conventional antifungal therapy for the treatment of denture stomatitis: A randomized clinical trial. Clin. Microbiol. Infect..

[176] Hosny K.M., Aldawsari H.M., Bahmdan R.H., Sindi A.M., Kurakula M., Alrobaian M.M., Aldryhim A.Y., Alkhalidi H.M., Bahmdan H.H., Khallaf R.A. (2019). Preparation, optimization, and evaluation of hyaluronic acid-based hydrogel loaded with miconazole self-nanoemulsion for the treatment of oral thrush. AAPS PharmSciTech.

[177] Roque L., Alopaeus J., Reis C., Rijo P., Molpeceres J., Hagesaether E., Tho I., Reis C. (2018). Mucoadhesive assessment of different antifungal nanoformulations. Bioinspir. Biomim..

[178] Niemirowicz K., Durnas B., Tokajuk G., Piktel E., Michalak G., Gu X., Kulakowska A., Savage P.B., Bucki R. (2017). Formulation and candidacidal activity of magnetic nanoparticles coated with cathelicidin LL-37 and ceragenin CSA-13. Sci. Rep..

[179] de Carvalho F.G., Magalhaes T.C., Teixeira N.M., Gondim B.L.C., Carlo H.L., Dos Santos R.L., de Oliveira A.R., Denadai A.M.L. (2019). Synthesis and characterization of TPP/chitosan nanoparticles: Colloidal mechanism of reaction and antifungal effect on C. albicans biofilm formation. Mater. Sci. Eng. C Mater. Biol. Appl..

[180] Gondim B.L.C., Castellano L.R.C., de Castro R.D., Machado G., Carlo H.L., Valenca A.M.G., de Carvalho F.G. (2018). Effect of chitosan nanoparticles on the inhibition of Candida spp. biofilm on denture base surface. Arch. Oral Biol..

[181] Tan Y., Leonhard M., Moser D., Schneider-Stickler B. (2016). Antibiofilm activity of carboxymethyl chitosan on the biofilms of non-Candida albicans Candida species. Carbohydr. Polym..

[182] Tonglairoum P., Woraphatphadung T., Ngawhirunpat T., Rojanarata T., Akkaramongkolporn P., Sajomsang W., Opanasopit P. (2017). Development and evaluation of N-naphthyl-N,O-succinyl chitosan micelles containing clotrimazole for oral candidiasis treatment. Pharm. Dev. Technol..

[183] Fabio C.A., Yolanda M.B., Carmen G.M., Francisco C., Antonio Julian B., Leonor P.L., Jesus S. (2016). Use of photodynamic therapy and chitosan for inactivacion of Candida albicans in a murine model. J. Oral Pathol. Med..

[184] Sakima V.T., Barbugli P.A., Cerri P.S., Chorilli M., Carmello J.C., Pavarina A.C., Mima E.G.O. (2018). Antimicrobial photodynamic therapy mediated by curcumin-loaded polymeric nanoparticles in a murine model of oral Candidiasis. Molecules.

[185] Cabrini Carmello J., Alves F., Basso F.G., de Souza Costa C.A., Tedesco A.C., Lucas Primo F., Mima E.G.O., Pavarina A.C. (2019). Antimicrobial photodynamic therapy reduces adhesion capacity and biofilm formation of Candida albicans from induced oral candidiasis in mice. Photodiagn. Photodyn. Ther..

[186] Mahattanadul S., Mustafa M.W., Kuadkaew S., Pattharachayakul S., Ungphaiboon S., Sawanyawisuth K. (2018). Oral ulcer healing and anti-Candida efficacy of an alcohol-free chitosan-curcumin mouthwash. Eur. Rev. Med. Pharmacol. Sci..

[187] Mustafa M.W., Ungphaiboon S., Phadoongsombut N., Pangsomboon K., Chelae S., Mahattanadul S. (2019). Effectiveness of an alcohol-free chitosan-curcuminoid mouthwash compared with chlorhexidine mouthwash in denture stomatitis treatment: A randomized trial. J. Altern. Complement. Med..

[188] Tejada G., Piccirilli G.N., Sortino M., Salomon C.J., Lamas M.C., Leonardi D. (2017). Formulation and in-vitro efficacy of antifungal mucoadhesive polymeric matrices for the delivery of miconazole nitrate. Mater. Sci. Eng. C Mater. Biol. Appl..

[189] Tejada G., Barrera M.G., Piccirilli G.N., Sortino M., Frattini A., Salomon C.J., Lamas M.C., Leonardi D. (2017). Development and evaluation of buccal films based on chitosan for the potential treatment of oral candidiasis. AAPS PharmSciTech.

[190] Kenechukwu F.C., Attama A.A., Ibezim E.C. (2017). Novel solidified reverse micellar solution-based mucoadhesive nano lipid gels encapsulating miconazole nitrate-loaded nanoparticles for improved treatment of oropharyngeal candidiasis. J. Microencapsul..

[191] Zhang P., Yang X., He Y., Chen Z., Liu B., Emesto C.S., Yang G., Wang W., Zhang J., Lin R. (2017). Preparation, characterization and toxicity evaluation of amphotericin B loaded MPEG-PCL micelles and its application for buccal tablets. Appl. Microbiol. Biotechnol..

[192] Rencber S., Karavana S.Y., Yilmaz F.F., Erac B., Nenni M., Ozbal S., Pekcetin C., Gurer-Orhan H., Hosgor-Limoncu M., Guneri P. (2016). Development, characterization, and in vivo assessment of mucoadhesive nanoparticles containing fluconazole for the local treatment of oral candidiasis. Int. J. Nanomed..

[193] Niyogi P., Pattnaik S., Maharana L., Mohapatra R., Haldar S. (2020). Temperature-dependent mucosal permeation kinetics of stigmasterol microspheres: In vivo mice model antioral candidiasis study. J. Biomed. Mater. Res. B Appl. Biomater..

[194] Roque L., Duarte N., Bronze M.R., Garcia C., Alopaeus J., Molpeceres J., Hagesaether E., Tho I., Rijo P., Reis C. (2018). Development of a bioadhesive nanoformulation with Glycyrrhiza glabra L. extract against Candida albicans. Biofouling.

[195] Lee H.L., Wang R.S., Hsu Y.C., Chuang C.C., Chan H.R., Chiu H.C., Wang Y.B., Chen K.Y., Fu E. (2018). Antifungal effect of tissue conditioners containing poly(acryloyloxyethyltrimethyl ammonium chloride)-grafted chitosan on Candida albicans growth in vitro. J. Dent. Sci..

[196] Mousavi S.A., Ghotaslou R., Kordi S., Khoramdel A., Aeenfar A., Kahjough S.T., Akbarzadeh A. (2018). Antibacterial and antifungal effects of chitosan nanoparticles on tissue conditioners of complete dentures. Int. J. Biol. Macromol..

[197] Saeed A., Haider A., Zahid S., Khan S.A., Faryal R., Kaleem M. (2019). In-vitro antifungal efficacy of tissue conditioner-chitosan composites as potential treatment therapy for denture stomatitis. Int. J. Biol. Macromol..

[198] Mousavi S.A., Ghotaslou R., Akbarzadeh A., Azima N., Aeinfar A., Khorramdel A. (2019). Evaluation of antibacterial and antifungal properties of a tissue conditioner used in complete dentures after incorporation of ZnOAg nanoparticles. J. Dent. Res. Dent. Clin. Dent. Prospects.

[199] Herla M., Boening K., Meissner H., Walczak K. (2019). Mechanical and surface properties of resilient denture liners modified with chitosan salts. Materials.

[200] Namangkalakul W., Benjavongkulchai S., Pochana T., Promchai A., Satitviboon W., Howattanapanich S., Phuprasong R., Ungvijanpunya N., Supakanjanakanti D., Chaitrakoonthong T. (2020). Activity of chitosan antifungal denture adhesive against common Candida species and Candida albicans adherence on denture base acrylic resin. J. Prosthet. Dent..

[201] Zambom C.R., da Fonseca F.H., Crusca E., da Silva P.B., Pavan F.R., Chorilli M., Garrido S.S. (2019). A novel antifungal system with potential for prolonged delivery of histatin 5 to limit growth of Candida albicans. Front. Microbiol..

[202] Park S.C., Kim Y.M., Lee J.K., Kim N.H., Kim E.J., Heo H., Lee M.Y., Lee J.R., Jang M.K. (2017). Targeting and synergistic action of an antifungal peptide in an antibiotic drug-delivery system. J. Control. Release.

[203] Mariadoss A.V.A., Vinayagam R., Senthilkumar V., Paulpandi M., Murugan K., Xu B., Gothandam K.M., Kotakadi V.S., David E. (2019). Phloretin loaded chitosan nanoparticles augments the pH-dependent mitochondrial-mediated intrinsic apoptosis in human oral cancer cells. Int. J. Biol. Macromol..

[204] Gao A., Teng Y., Seyiti P., Yen Y., Qian H., Xie C., Li R., Lin Z. (2019). Using omniscan-loaded nanoparticles as a tumor-targeted MRI contrast agent in oral squamous cell carcinoma by gelatinase-stimuli strategy. Nanoscale Res. Lett..

[205] Baldea I., Florea A., Olteanu D., Clichici S., David L., Moldovan B., Cenariu M., Achim M., Suharoschi R., Danescu S. (2020). Effects of silver and gold nanoparticles phytosynthesized with Cornus mas extract on oral dysplastic human cells. Nanomedicine.

[206] Mohan A., Narayanan S., Balasubramanian G., Sethuraman S., Krishnan U.M. (2016). Dual drug loaded nanoliposomal chemotherapy: A promising strategy for treatment of head and neck squamous cell carcinoma. Eur. J. Pharm. Biopharm..

[207] Suliman S., Mustafa K., Krueger A., Steinmüller-Nethl D., Finne-Wistrand A., Osdal T., Hamza A.O., Sun Y., Parajuli H., Waag T. (2016). Nanodiamond modified copolymer scaffolds affects tumour progression of early neoplastic oral keratinocytes. Biomaterials.

[208] Barua S., Banerjee P.P., Sadhu A., Sengupta A., Chatterjee S., Sarkar S., Barman S., Chattopadhyay A., Battacharya S., Mondal N.C. (2017). Silver nanoparticles as antibacterial and anticancer materials against human breast, cervical and oral cancer cells. J. Nanosci. Nanotechnol..

[209] Maheswari P., Harish S., Navaneethan M., Muthamizhchelvan C., Ponnusamy S., Hayakawa Y. (2020). Bio-modified TiO(2) nanoparticles with Withania somnifera, Eclipta prostrata and Glycyrrhiza glabra for anticancer and antibacterial applications. Mater. Sci. Eng. C Mater. Biol. Appl..

[210] Mehnath S., Arjama M., Rajan M., Annamalai G., Jeyaraj M. (2018). Co-encapsulation of dual drug loaded in MLNPs: Implication on sustained drug release and effectively inducing apoptosis in oral carcinoma cells. Biomed. Pharmacother..

[211] Neshastehriz A., Tabei M., Maleki S., Eynali S., Shakeri-Zadeh A. (2017). Photothermal therapy using folate conjugated gold nanoparticles enhances the effects of 6MV X-ray on mouth epidermal carcinoma cells. J. Photochem. Photobiol. B.

[212] Poojari R., Kini S., Srivastava R., Panda D. (2016). Intracellular interactions of electrostatically mediated layer-by-layer assembled polyelectrolytes based sorafenib nanoparticles in oral cancer cells. Colloids Surf. B Biointerfaces.

[213] Bonan R.F., Mota M.F., da Costa Farias R.M., da Silva S.D., Bonan P.R.F., Diesel L., Menezes R.R., da Cruz Perez D.E. (2019). In vitro antimicrobial and anticancer properties of TiO(2) blow-spun nanofibers containing silver nanoparticles. Mater. Sci. Eng. C Mater. Biol. Appl..

[214] Furqan M., Huma Z., Ashfaq Z., Nasir A., Ullah R., Bilal A., Iqbal M., Khalid M.H., Hussain I., Faisal A. (2019). Identification and evaluation of novel drug combinations of Aurora kinase inhibitor CCT137690 for enhanced efficacy in oral cancer cells. Cell Cycle.

[215] Cacciotti I., Chronopoulou L., Palocci C., Amalfitano A., Cantiani M., Cordaro M., Lajolo C., Callà C., Boninsegna A., Lucchetti D. (2018). Controlled release of 18-β-glycyrrhetic acid by nanodelivery systems increases cytotoxicity on oral carcinoma cell line. Nanotechnology.

[216] Yang L.X., Wu Y.N., Wang P.W., Su W.C., Shieh D.B. (2019). Iron release profile of silica-modified zero-valent iron NPs and their implication in cancer therapy. Int. J. Mol. Sci..

[217] Moosavi Nejad S., Takahashi H., Hosseini H., Watanabe A., Endo H., Narihira K., Kikuta T., Tachibana K. (2016). Acute effects of sono-activated photocatalytic titanium dioxide nanoparticles on oral squamous cell carcinoma. Ultrason. Sonochem..

[218] Nam S., Lee S.Y., Cho H.J. (2017). Phloretin-loaded fast dissolving nanofibers for the locoregional therapy of oral squamous cell carcinoma. J. Colloid. Interface Sci..

[219] Lai K.C., Chueh F.S., Hsiao Y.T., Cheng Z.Y., Lien J.C., Liu K.C., Peng S.F., Chung J.G. (2019). Gefitinib and curcumin-loaded nanoparticles enhance cell apoptosis in human oral cancer SAS cells in vitro and inhibit SAS cell xenografted tumor in vivo. Toxicol. Appl. Pharmacol..

[220] Murata T., Kutsuna T., Kurohara K., Shimizu K., Tomeoku A., Arai N. (2018). Evaluation of a new hydroxyapatite nanoparticle as a drug delivery system to oral squamous cell carcinoma cells. Anticancer Res..

[221] Jin B.Z., Dong X.Q., Xu X., Zhang F.H. (2018). Development and in vitro evaluation of mucoadhesive patches of methotrexate for targeted delivery in oral cancer. Oncol. Lett..

[222] Wei Z., Yin X., Cai Y., Xu W., Song C., Wang Y., Zhang J., Kang A., Wang Z., Han W. (2018). Antitumor effect of a Pt-loaded nanocomposite based on graphene quantum dots combats hypoxia-induced chemoresistance of oral squamous cell carcinoma. Int. J. Nanomed..

[223] Jin L., Wang Q., Chen J., Wang Z., Xin H., Zhang D. (2019). Efficient delivery of therapeutic siRNA by Fe(3)O(4) magnetic nanoparticles into oral cancer cells. Pharmaceutics.

[224] Lang L., Lam T., Chen A., Jensen C., Duncan L., Kong F.C., Kurago Z.B., Shay C., Teng Y. (2020). Circumventing AKT-associated radioresistance in oral cancer by novel nanoparticle-encapsulated capivasertib. Cells.

[225] Li X., Li L., Huang Y., Liu B., Chi H., Shi L., Zhang W., Li G., Niu Y., Zhu X. (2017). Synergistic therapy of chemotherapeutic drugs and MTH1 inhibitors using a pH-sensitive polymeric delivery system for oral squamous cell carcinoma. Biomater. Sci..

[226] Shi X.L., Li Y., Zhao L.M., Su L.W., Ding G. (2019). Delivery of MTH1 inhibitor (TH287) and MDR1 siRNA via hyaluronic acid-based mesoporous silica nanoparticles for oral cancers treatment. Colloids Surf. B Biointerfaces.

[227] Wang J., Gao S., Wang S., Xu Z., Wei L. (2018). Zinc oxide nanoparticles induce toxicity in CAL 27 oral cancer cell lines by activating PINK1/Parkin-mediated mitophagy. Int. J. Nanomed..

[228] Kowshik J., Nivetha R., Ranjani S., Venkatesan P., Selvamuthukumar S., Veeravarmal V., Nagini S. (2019). Astaxanthin inhibits hallmarks of cancer by targeting the PI3K/NF-κΒ/STAT3 signalling axis in oral squamous cell carcinoma models. IUBMB Life.

[229] Legge C.J., Colley H.E., Lawson M.A., Rawlings A.E. (2019). Targeted magnetic nanoparticle hyperthermia for the treatment of oral cancer. J. Oral Pathol. Med..

[230] Nayak A., Siddharth S., Das S., Nayak D., Sethy C., Kundu C.N. (2017). Nanoquinacrine caused apoptosis in oral cancer stem cells by disrupting the interaction between GLI1 and β catenin through activation of GSK3β. Toxicol. Appl. Pharmacol..

[231] Satapathy S.R., Nayak A., Siddharth S., Das S., Nayak D., Kundu C.N. (2018). Metallic gold and bioactive quinacrine hybrid nanoparticles inhibit oral cancer stem cell and angiogenesis by deregulating inflammatory cytokines in p53 dependent manner. Nanomedicine.

[232] Gupta P., Singh M., Kumar R., Belz J., Shanker R., Dwivedi P.D., Sridhar S., Singh S.P. (2018). Synthesis and in vitro studies of PLGA-DTX nanoconjugate as potential drug delivery vehicle for oral cancer. Int. J. Nanomed..

[233] Hembram K.C., Chatterjee S., Sethy C., Nayak D., Pradhan R., Molla S., Bindhani B.K., Kundu C.N. (2019). Comparative and mechanistic study on the anticancer activity of quinacrine-based silver and gold hybrid nanoparticles in head and neck cancer. Mol. Pharm..

[234] Matos B.N., Pereira M.N., Bravo M.O., Cunha-Filho M., Saldanha-Araújo F., Gratieri T., Gelfuso G.M. (2020). Chitosan nanoparticles loading oxaliplatin as a mucoadhesive topical treatment of oral tumors: Iontophoresis further enhances drug delivery ex vivo. Int. J. Biol. Macromol..

[235] Dziedzic A., Kubina R., Bułdak R.J., Skonieczna M., Cholewa K. (2016). Silver nanoparticles exhibit the dose-dependent anti-proliferative effect against human squamous carcinoma cells attenuated in the presence of berberine. Molecules.

[236] Srivastava S., Gupta S., Mohammad S., Ahmad I. (2019). Development of α-tocopherol surface-modified targeted delivery of 5-fluorouracil-loaded poly-D, L-lactic-co-glycolic acid nanoparticles against oral squamous cell carcinoma. J. Cancer Res. Ther..

[237] Srivastava S., Mohammad S., Pant A.B., Mishra P.R., Pandey G., Gupta S., Farooqui S. (2018). Co-delivery of 5-fluorouracil and curcumin nanohybrid formulations for improved chemotherapy against oral squamous cell carcinoma. J. Maxillofac. Oral Surg..

[238] Rathinaraj P., Muthusamy G., Prasad N.R., Gunaseelan S., Kim B., Zhu S. (2020). Folate-gold-bilirubin nanoconjugate induces apoptotic death in multidrug-resistant oral carcinoma cells. Eur. J. Drug Metab. Pharmacokinet..

[239] El-Hamid E.S.A., Gamal-Eldeen A.M., Sharaf Eldeen A.M. (2019). Liposome-coated nano doxorubicin induces apoptosis on oral squamous cell carcinoma CAL-27 cells. Arch. Oral Biol..

[240] Bharadwaj R., Sahu B.P., Haloi J., Laloo D., Barooah P., Keppen C., Deka M., Medhi S. (2019). Combinatorial therapeutic approach for treatment of oral squamous cell carcinoma. Artif. Cells Nanomed. Biotechnol..

[241] Shtenberg Y., Goldfeder M., Prinz H., Shainsky J., Ghantous Y., Abu El-Naaj I., Schroeder A., Bianco-Peled H. (2018). Mucoadhesive alginate pastes with embedded liposomes for local oral drug delivery. Int. J. Biol. Macromol..

[242] Moradzadeh Khiavi M., Anvari E., Hamishehkar H., Abdal K. (2019). Assessment of the blood parameters, cardiac and liver Enzymes in oral squamous cell carcinoma following treated with injectable doxorubicin-loaded nano-particles. Asian Pac. J. Cancer Prev..

[243] Li Q., Wen Y., You X., Zhang F., Shah V., Chen X., Tong D., Wei X., Yin L., Wu J. (2016). Development of a reactive oxygen species (ROS)-responsive nanoplatform for targeted oral cancer therapy. J. Mater. Chem. B.

[244] Wang Y., Wan G., Li Z., Shi S., Chen B., Li C., Zhang L., Wang Y. (2017). PEGylated doxorubicin nanoparticles mediated by HN-1 peptide for targeted treatment of oral squamous cell carcinoma. Int. J. Pharm..

[245] Shi S., Zhang L., Zhu M., Wan G., Li C., Zhang J., Wang Y., Wang Y. (2018). Reactive oxygen species-responsive nanoparticles based on peglated prodrug for targeted treatment of oral tongue squamous cell carcinoma by combining photodynamic therapy and chemotherapy. ACS Appl. Mater. Interfaces.

[246] Wang D., Xu X., Zhang K., Sun B., Wang L., Meng L., Liu Q., Zheng C., Yang B., Sun H. (2018). Codelivery of doxorubicin and MDR1-siRNA by mesoporous silica nanoparticles-polymerpolyethylenimine to improve oral squamous carcinoma treatment. Int. J. Nanomed..

[247] Xiong J., Feng J., Qiu L., Gao Z., Li P., Pang L., Zhang Z. (2019). SDF-1-loaded PLGA nanoparticles for the targeted photoacoustic imaging and photothermal therapy of metastatic lymph nodes in tongue squamous cell carcinoma. Int. J. Pharm..

[248] Liu Z., Shi J., Zhu B., Xu Q. (2020). Development of a multifunctional gold nanoplatform for combined chemo-photothermal therapy against oral cancer. Nanomedicine.

[249] Ankri R., Ashkenazy A., Milstein Y., Brami Y., Olshinka A., Goldenberg-Cohen N., Popovtzer A., Fixler D., Hirshberg A. (2016). Gold nanorods based air scanning electron microscopy and diffusion reflection imaging for mapping tumor margins in squamous cell carcinoma. ACS Nano.

[250] Chakraborty D., Viveka T.S., Arvind K., Shyamsundar V., Kanchan M., Alex S.A., Chandrasekaran N., Vijayalakshmi R., Mukherjee A. (2018). A facile gold nanoparticle-based ELISA system for detection of osteopontin in saliva: Towards oral cancer diagnostics. Clin. Chim. Acta.

[251] Kim C.S., Ingato D., Wilder-Smith P., Chen Z., Kwon Y.J. (2018). Stimuli-disassembling gold nanoclusters for diagnosis of early stage oral cancer by optical coherence tomography. Nano Converg..

[252] Tan Y., Yan B., Xue L., Li Y., Luo X., Ji P. (2017). Surface-enhanced Raman spectroscopy of blood serum based on gold nanoparticles for the diagnosis of the oral squamous cell carcinoma. Lipids Health Dis..

[253] Xue L., Yan B., Li Y., Tan Y., Luo X., Wang M. (2018). Surface-enhanced Raman spectroscopy of blood serum based on gold nanoparticles for tumor stages detection and histologic grades classification of oral squamous cell carcinoma. Int. J. Nanomed..

[254] Fălămaș A., Rotaru H., Hedeșiu M. (2020). Surface-enhanced Raman spectroscopy (SERS) investigations of saliva for oral cancer diagnosis. Lasers Med. Sci..

[255] Verma S., Singh A., Shukla A., Kaswan J., Arora K., Ramirez-Vick J., Singh P., Singh S.P. (2017). Anti-IL8/AuNPs-rGO/ITO as an Immunosensing Platform for Noninvasive Electrochemical Detection of Oral Cancer. ACS Appl. Mater. Interfaces.

[256] Yang Y., Zhou B., Zhou J., Shi X., Sha Y., Wu H. (2018). Assessment of lingual sentinel lymph nodes metastases using dual-modal indirect CT/MR lymphography with gold-gadolinium-based nanoprobes in a tongue VX(2) carcinoma model. Acta Otolaryngol..

[257] Chan Y.C., Chen C.W., Chan M.H., Chang Y.C., Chang W.M., Chi L.H., Yu H.M., Lin Y.F., Tsai D.P., Liu R.S. (2016). MMP2-sensing up-conversion nanoparticle for fluorescence biosensing in head and neck cancer cells. Biosens. Bioelectron..

[258] Wu W.J., Zheng L., Zhang J.G. (2019). The use of carbon nanoparticles to track occult lingual lymph nodes in early-stage tongue squamous cell carcinoma. Int. J. Oral Maxillofac. Surg..

[259] Moisoiu V., Stefancu A., Gulei D., Boitor R., Magdo L., Raduly L., Pasca S., Kubelac P., Mehterov N., Chiș V. (2019). SERS-based differential diagnosis between multiple solid malignancies: Breast, colorectal, lung, ovarian and oral cancer. Int. J. Nanomed..

[260] Kumar S., Sharma J.G., Maji S., Malhotra B.D. (2016). Nanostructured zirconia decorated reduced graphene oxide based efficient biosensing platform for non-invasive oral cancer detection. Biosens. Bioelectron..

[261] Chundayil Madathil G., Iyer S., Thankappan K., Gowd G.S., Nair S., Koyakutty M. (2019). A novel surface enhanced Raman catheter for rapid detection, classification, and grading of oral cancer. Adv. Healthc. Mater..

[262] Kumar S., Panwar S., Kumar S., Augustine S., Malhotra B.D. (2019). Biofunctionalized nanostructured yttria modified non-invasive impedometric biosensor for efficient detection of oral cancer. Nanomaterials.

[263] Lang L., Shay C., Zhao X., Xiong Y., Wang X., Teng Y. (2019). Simultaneously inactivating Src and AKT by saracatinib/capivasertib co-delivery nanoparticles to improve the efficacy of anti-Src therapy in head and neck squamous cell carcinoma. J. Hematol. Oncol..

[264] Su Z., Liu D., Chen L., Zhang J., Ru L., Chen Z., Gao Z., Wang X. (2019). CD44-targeted magnetic nanoparticles kill head and neck squamous cell carcinoma stem cells in an alternating magnetic field. Int. J. Nanomed..

[265] Mizrachi A., Shamay Y., Shah J., Brook S., Soong J., Rajasekhar V.K., Humm J.L., Healey J.H., Powell S.N., Baselga J. (2017). Tumour-specific PI3K inhibition via nanoparticle-targeted delivery in head and neck squamous cell carcinoma. Nat. Commun..

[266] Damiani V., Falvo E., Fracasso G., Federici L., Pitea M., De Laurenzi V., Sala G., Ceci P. (2017). Therapeutic efficacy of the novel stimuli-sensitive nano-ferritins containing doxorubicin in a head and neck cancer model. Int. J. Mol. Sci..

[267] Zhang Z., Zhuang L., Lin Y., Yan M., Lv J., Li X., Lin H., Zhu P., Lin Q., Xu Y. (2020). Novel drug delivery system based on hollow mesoporous magnetic nanoparticles for head and neck cancers--targeted therapy in vitro and in vivo. Am. J. Cancer Res..

[268] Lang L., Shay C., Xiong Y., Thakkar P., Chemmalakuzhy R., Wang X., Teng Y. (2018). Combating head and neck cancer metastases by targeting Src using multifunctional nanoparticle-based saracatinib. J. Hematol. Oncol..

[269] Li H., Shi L., Wei J., Zhang C., Zhou Z., Wu L., Liu W. (2016). Cellular uptake and anticancer activity of salvianolic acid B phospholipid complex loaded nanoparticles in head and neck cancer and precancer cells. Colloids Surf. B Biointerfaces.

[270] Teraoka S., Kakei Y., Akashi M., Iwata E., Hasegawa T., Miyawaki D., Sasaki R., Komori T. (2018). Gold nanoparticles enhance X-ray irradiation-induced apoptosis in head and neck squamous cell carcinoma in vitro. Biomed. Rep..

[271] Lecaros R.L., Huang L., Lee T.C., Hsu Y.C. (2016). Nanoparticle delivered VEGF-A siRNA enhances photodynamic therapy for head and neck cancer treatment. Mol. Ther..

[272] Takeuchi I., Kamiki Y., Makino K. (2018). Therapeutic efficacy of rebamipide-loaded PLGA nanoparticles coated with chitosan in a mouse model for oral mucositis induced by cancer chemotherapy. Colloids Surf. B Biointerfaces.

[273] Choi J.S., Han S.H., Hyun C., Yoo H.S. (2016). Buccal adhesive nanofibers containing human growth hormone for oral mucositis. J. Biomed. Mater. Res. B Appl. Biomater..

[274] Takeuchi I., Togo C., Makino K. (2019). Rebamipide-containing film using chitosan and HPMC for oral mucositis induced by cancer chemotherapy. Anticancer Res..

[275] Nam K., Dean S.M., Brown C.T., Smith R.J., Lei P., Andreadis S.T., Baker O.J. (2019). Synergistic effects of laminin-1 peptides, VEGF and FGF9 on salivary gland regeneration. Acta Biomater..

[276] Nam K., Maruyama C.L., Wang C.S., Trump B.G., Lei P., Andreadis S.T., Baker O.J. (2017). Laminin-111-derived peptide conjugated fibrin hydrogel restores salivary gland function. PLoS ONE.

[277] Villa A., Connell C.L., Abati S. (2015). Diagnosis and management of xerostomia and hyposalivation. Ther. Clin. Risk Manag..

[278] Escobar A., Aitken J. (2019). Xerostomia: An update of causes and treatments. Salivary Glands-New Approaches in Diagnostics and Treatment.

[279] Adamczak M.I., Martinsen Ø.G., Smistad G., Hiorth M. (2016). Water sorption properties of HM-pectin and liposomes intended to alleviate dry mouth. Int. J. Pharm..

[280] Adamczak M.I., Hagesaether E., Smistad G., Hiorth M. (2016). An in vitro study of mucoadhesion and biocompatibility of polymer coated liposomes on HT29-MTX mucus-producing cells. Int. J. Pharm..

[281] Adamczak M.I., Martinsen Ø.G., Smistad G., Hiorth M. (2017). Polymer coated mucoadhesive liposomes intended for the management of xerostomia. Int. J. Pharm..

[282] Tran P.H.L., Duan W., Tran T.T.D. (2019). Recent developments of nanoparticle-delivered dosage forms for buccal delivery. Int. J. Pharm..

[283] Hua S. (2019). Advances in nanoparticulate drug delivery approaches for sublingual and buccal administration. Front. Pharmacol..

[284] Al-Dhubiab B.E., Nair A.B., Kumria R., Attimarad M., Harsha S. (2016). Development and evaluation of buccal films impregnated with selegiline-loaded nanospheres. Drug Deliv..

[285] Al-Dhubiab B.E. (2016). In vitro and in vivo evaluation of nano-based films for buccal delivery of zolpidem. Braz. Oral Res..

[286] Castro P.M., Baptista P., Madureira A.R., Sarmento B., Pintado M.E. (2018). Combination of PLGA nanoparticles with mucoadhesive guar-gum films for buccal delivery of antihypertensive peptide. Int. J. Pharm..

[287] Abd El Azim H., Nafee N., Ramadan A., Khalafallah N. (2015). Liposomal buccal mucoadhesive film for improved delivery and permeation of water-soluble vitamins. Int. J. Pharm..

[288] Mašek J., Lubasová D., Lukáč R., Turánek-Knotigová P., Kulich P., Plocková J., Mašková E., Procházka L., Koudelka Š., Sasithorn N. (2017). Multi-layered nanofibrous mucoadhesive films for buccal and sublingual administration of drug-delivery and vaccination nanoparticles-important step towards effective mucosal vaccines. J. Control. Release.

[289] Al-Nemrawi N.K., Alsharif S.S.M., Alzoubi K.H., Alkhatib R.Q. (2019). Preparation and characterization of insulin chitosan-nanoparticles loaded in buccal films. Pharm. Dev. Technol..

[290] Kraisit P., Limmatvapirat S., Luangtana-Anan M., Sriamornsak P. (2018). Buccal administration of mucoadhesive blend films saturated with propranolol loaded nanoparticles. Asian J. Pharm. Sci..

[291] Castro P.M., Baptista P., Zuccheri G., Madureira A.R., Sarmento B., Pintado M.E. (2019). Film-nanoparticle composite for enhanced oral delivery of alpha-casozepine. Colloids Surf. B Biointerfaces.

[292] Abozaid D., Ramadan A., Barakat H., Khalafallah N. (2018). Acyclovir lipid nanocapsules gel for oromucosal delivery: A preclinical evidence of efficacy in the chicken pouch membrane model. Eur. J. Pharm. Sci..

[293] Marques A.C., Rocha A.I., Leal P., Estanqueiro M., Lobo J.M.S. (2017). Development and characterization of mucoadhesive buccal gels containing lipid nanoparticles of ibuprofen. Int. J. Pharm..

[294] Muniz B.V., Baratelli D., Di Carla S., Serpe L., da Silva C.B., Guilherme V.A., Ribeiro L.N.M., Cereda C.M.S., de Paula E., Volpato M.C. (2018). Hybrid hydrogel composed of polymeric nanocapsules co-loading lidocaine and prilocaine for topical intraoral anesthesia. Sci. Rep..

[295] Mahdizadeh Barzoki Z., Emam-Djomeh Z., Mortazavian E., Akbar Moosavi-Movahedi A., Rafiee Tehrani M. (2016). Formulation, in vitro evaluation and kinetic analysis of chitosan-gelatin bilayer muco-adhesive buccal patches of insulin nanoparticles. J. Microencapsul..

[296] Hazzah H.A., Farid R.M., Nasra M.M., El-Massik M.A., Abdallah O.Y. (2015). Lyophilized sponges loaded with curcumin solid lipid nanoparticles for buccal delivery: Development and characterization. Int. J. Pharm..

[297] El-Nahas A.E., Allam A.N., El-Kamel A.H. (2017). Mucoadhesive buccal tablets containing silymarin Eudragit-loaded nanoparticles: Formulation, characterisation and ex vivo permeation. J. Microencapsul..

[298] Mura P., Cirri M., Mennini N., Casella G., Maestrelli F. (2016). Polymeric mucoadhesive tablets for topical or systemic buccal delivery of clonazepam: Effect of cyclodextrin complexation. Carbohydr. Polym..

